# Selective gene dosage by CRISPR‐Cas9 genome editing in hexaploid *Camelina sativa*

**DOI:** 10.1111/pbi.12671

**Published:** 2017-04-01

**Authors:** Céline Morineau, Yannick Bellec, Frédérique Tellier, Lionel Gissot, Zsolt Kelemen, Fabien Nogué, Jean‐Denis Faure

**Affiliations:** ^1^ Institut Jean‐Pierre Bourgin (IJPB)INRAAgroParisTechCNRSSaclay Plant Sciences (SPS)Université Paris‐SaclayVersaillesFrance

**Keywords:** CRISPR‐Cas9, oleic acid, Camelina, FAD2

## Abstract

In many plant species, gene dosage is an important cause of phenotype variation. Engineering gene dosage, particularly in polyploid genomes, would provide an efficient tool for plant breeding. The hexaploid oilseed crop *Camelina sativa,* which has three closely related expressed subgenomes, is an ideal species for investigation of the possibility of creating a large collection of combinatorial mutants. Selective, targeted mutagenesis of the three delta‐12‐desaturase (*FAD2*) genes was achieved by CRISPR‐Cas9 gene editing, leading to reduced levels of polyunsaturated fatty acids and increased accumulation of oleic acid in the oil. Analysis of mutations over four generations demonstrated the presence of a large variety of heritable mutations in the three isologous *CsFAD2* genes. The different combinations of single, double and triple mutants in the T3 generation were isolated, and the complete loss‐of‐function mutants revealed the importance of delta‐12‐desaturation for Camelina development. Combinatorial association of different alleles for the three *FAD2* loci provided a large diversity of Camelina lines with various lipid profiles, ranging from 10% to 62% oleic acid accumulation in the oil. The different allelic combinations allowed an unbiased analysis of gene dosage and function in this hexaploid species, but also provided a unique source of genetic variability for plant breeding.

## Introduction

Plant breeding is based on the recombination of a diversity of alleles to select the optimal combinations for a defined trait. Classical breeding relies on the availability of alleles in natural accessions, but also in the different subgenomes in the case of polyploid crop species. These limitations could be overcome by genetic modifications that allow the dominant expression of a transgene carrying the desired trait or allele, or alternatively the dominant down‐regulation of endogenous genes by silencing. Down‐regulation strategies, albeit efficient, may pose problems in the long run since the endogenous genes are still expressed, leading to potential instability of the desired trait after several generations (Rajeevkumar *et al*., 2015). Recently, the development of genome‐editing strategies allowed selective site‐directed mutagenesis even in large polyploid genomes, providing a very attractive approach to creating new stable alleles in crops.

Genome editing is based on the creation of one or more breaks in the genome DNA at a specific localization. Double‐strand breaks undergo repair either by error‐prone nonhomologous end joining (NHEJ) or by high‐fidelity homologous recombination that requires the presence of a template, usually provided by the other chromosomal strand. Imperfect repair of the DNA break leads, at a variable frequency, to nucleotide addition or deletion, thereby creating a stable mutation. The creation of specific DNA breaks can be obtained by the expression of nucleases such as zinc‐finger nucleases (ZFNs), transcription activator‐like effector nucleases (TALENs) and, more recently, the Cas9 protein associated with type II clustered regulatory interspaced short palindromic repeats (CRISPR) (Hsu *et al*., 2014). CRISPR‐Cas9 relies on the presence of a 20‐nucleotide guide RNA (sgRNA) that targets specifically the Cas9 nuclease to the complementary genomic sequence (Jinek *et al*., 2012). The sgRNA harbours the so‐called ‘protospacer adjacent motif’ (PAM), for which the consensus sequence, NGG, is adjacent to the 3′ end of the 20‐bp target. The specificity of the system relies on the perfect match of 8–12 nucleotides of the 5′ end of the target, while some mismatches are tolerated at the 3′ end (Cong *et al*., 2013). This system offers a simple, rapid and flexible method to induce single or even multiple mutations in the genome, defined by the specificity of the selected sgRNA sequence.

The CRISPR‐Cas9 system was first validated by transient expression in several species including Arabidopsis, rice, tobacco (*Nicotiana benthamiana),* sorghum and sweet orange (Feng *et al*., 2013; Jia and Nian, 2014; Jiang *et al*., 2013; Li *et al*., 2013; Liang *et al*., 2014; Mao *et al*., 2013; Miao *et al*., 2013; Nekrasov *et al*., 2013; Shan *et al*., 2013; Xie and Yang, 2013). Highly efficient, stable mutagenesis was subsequently demonstrated in Arabidopsis, as well as in several crops: rice, wheat, barley, *Brassica oleracea*, potato, maize and soybean (Brooks *et al*., 2014; Butler *et al*., 2015; Čermák *et al*., 2015; Fauser *et al*., 2014; Feng *et al*., 2014; Ito *et al*., 2015; Jiang *et al*., 2014; Lawrenson *et al*., 2015; Li *et al*., 2015; Svitashev *et al*., 2015; Wang *et al*., 2014; Zhang *et al*., 2014; Zhou *et al*., 2014). The CRISPR‐Cas9 system can be used to achieve multiple mutagenesis by the expression of several sgRNAs targetting different genes (Lowder *et al*., 2015; Ma *et al*., 2015). The versatility and efficiency of CRISPR‐Cas9 system were also improved by Golden Braid and Gateway‐based vectors (Ma *et al*., 2015; Vazquez‐Vilar *et al*., 2016) and germ‐line‐specific Cas9 expression (Mao *et al*., 2016). One major challenge is the ability of CRISPR‐Cas9 to induce mutations in polyploid genomes, in particular those with closely related subgenomes. CRISPR‐Cas9 was effective in diploid and tetraploid potato, tetraploid tobacco and hexaploid wheat (Butler *et al*., 2015; Gao *et al*., 2014; Wang *et al*., 2014). The presence of mutations in the different homeologous genes was only assessed in wheat, but the low number of regenerated mutants prevented accurate estimation of the type of alleles obtained as well as their frequency (Wang *et al*., 2014).

To investigate the use of CRISPR‐Cas9 to generate a large diversity of allele combinations in a polyploid species, we used *Camelina sativa*, also known as false flax, an oilseed crop in the *Brassicaceae* family that is closely related to *Arabidopsis thaliana* (Al‐Shehbaz *et al*., 2006; Haslam *et al*., 2016). The Camelina genome is composed of three subgenomes, two of which are extremely similar to each other and may be derived from an event of autopolyploidy, while the third one is slightly more divergent (Kagale *et al*., 2014). Subgenome‐specific transcriptomic studies showed a remarkably low degree of gene loss and gene functional differentiation among the three subgenomes (Kagale *et al*., 2014, 2016; Mudalkar *et al*., 2014; Nguyen *et al*., 2013). The different homeologous genes were most often expressed with a slight enhanced expression level for genes from the most divergent subgenome (Kagale *et al*., 2014, 2016). Obtaining recessive mutants in Camelina is therefore limited by the high genetic redundancy. CRISPR‐Cas9 mutagenesis would therefore be of great interest to increase genetic diversity by obtaining multiple alleles in specific genes, but also to evaluate the effects of gene dosage on specific traits. An added value of Camelina is the ease of its transformation by floral dip, like for Arabidopsis, thus limiting the occurrence of somaclonal mutations caused by *in vitro* culture and regeneration (Lu and Kang, 2008). Camelina has a high level of polyunsaturated fatty acids in its oil, and several strategies have already been used to develop monounsaturated lines rich in oleic acid (Kang *et al*., 2011; Nguyen *et al*., 2013). Oleic acid (cis‐9‐octadecenoic acid, C18:1) is desaturated to linoleic acid (cis‐9,12‐octadecadienoic acid, C18:2) by the microsomal oleate desaturase or delta‐12 desaturase FAD2 (EC 1.3.1.35). The polyploidy of the Camelina genome has limited the identification of *fad2* mutants, and only antisense or RNAi suppression of *fad2* expression was efficient for increasing the accumulation of oleic acid (Kang *et al*., 2011; Nguyen *et al*., 2013).

Here, we report CRISPR‐Cas9‐induced mutations in hexaploid Camelina by isolating new oleic acid delta‐12 desaturase *FAD2* alleles. Accumulation of mutations was monitored over three generations of CRISPR‐Cas9 expression, to eventually isolate all possible null allele combinations at the three homeologous *FAD2* loci and a broad range of lines accumulating oleic acid to different levels. Complete FAD2 loss of function led to important development defects, revealing the importance of polyunsaturated fatty acids in plants.

## Results

### Expression of two sgRNAs targeting the three FAD2 sequences generated a variable number and type of mutations

Two sgRNAs were initially designed from the Arabidopsis *FAD2* sequence using TEFOR website prediction, and comparison with the sequences of the three homeologous *FAD2* genes from *Camelina sativa* var. Céline, *CsFAD2‐1, CsFAD2‐2* and *CsFAD2‐3* showed they should also be targeted (Hutcheon *et al*., 2010; Kang *et al*., 2011). The sgRNA sequences were chosen based on the following: (i) TEFOR specificity score, (ii) the absence of predicted off‐target sites, including up to 4 mismatches (even near the PAM), (iii) their presence in the first half of the coding sequence and (iv) the strict sequence conservation within the three *CsFAD2* genes (Figures S1A‐B and S2). The sgRNA#1 and sgRNA#2 were cloned downstream of the Camelina U3 and U6 promoters, respectively, in a modified version of the pDE‐Cas9 vector (Fauser *et al*., 2014), expressing a DsRed cassette suitable for the selection of Camelina transformants (Nguyen *et al*., 2013) (Figure S1C). More than 118 transformants were selected, and leaf DNA was collected from the T1 lines at early flowering stage and used to sequence the *FAD2* genes with nonspecific primers to score the presence of mutations (Table 1).

**Table 1:** Number of *fad2* mutations in T1, T2 and T3 generations of Camelina CRISPR lines. The # indicates the number of plants used to generate the next generation

Guide RNA	T1	T1 Mutation (%)	#T1 plants	T2	T2 Mutation (%)	#T2 plants	T3	T3 Mutation (%)
sgRNA1	99	0	6*	53	37.7 (20/53)	–	–	–
sgRNA2	19	26 (5/19)	5	44	81.8 (36/44)	5	133	98.4 (131/133)

The star (*) indicates the 6 lines that were picked randomly.

**Figure 1:**
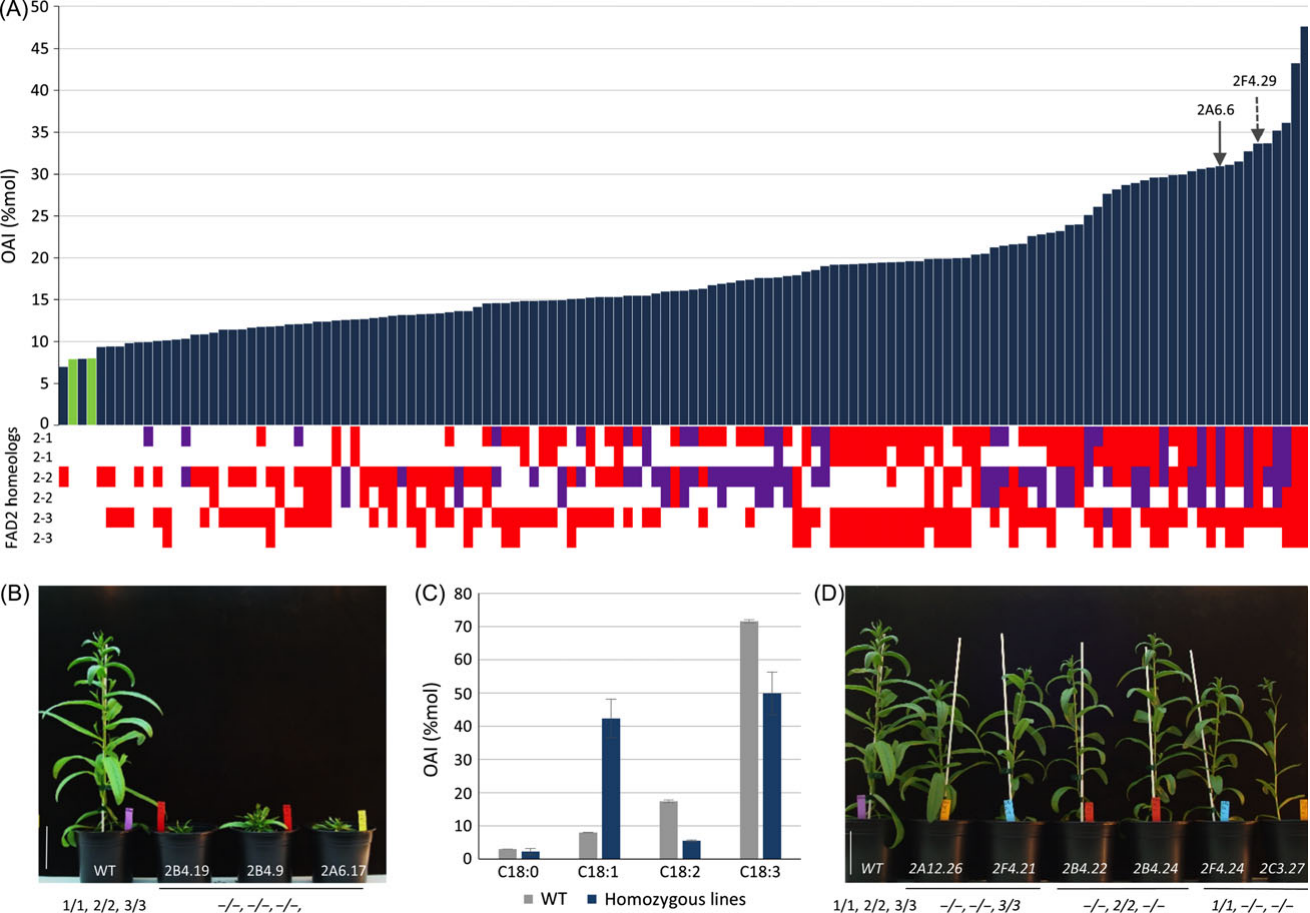
RNA‐guided Cas9 activity on Camelina *CsFAD2* genes modified oleic acid content and plant growth (A) Mutation occurrence at the *CsFAD2‐1, CsFAD2‐2* and *CsFAD2‐3* loci in individual T3 lines (bottom) and the resulting effect on oleic acid content measured as oleic acid index (OAI) of T3 leaves (top). Mutations leading to sequence frameshift are indicated in red, while those only associated with deletion or insertion without frameshift are indicated in purple. In green is indicated two wild‐type Camelina. An example of two lines carrying at *CsFAD2‐1* locus either a –TGG ^580^ deletion leading to W^194^∆ or a –G^579^ insertion (frameshift) is indicated with respectively a plain or a dashed arrow. (B) Phenotype of two‐month‐old triple homozygous *csfad2* mutants (2B4‐19, 2B4‐9, 2A6‐17) compared to wild type (WT). (C) OAI of leaves from wild type (grey) and triple *csfad2* mutants (blue). Data are the mean (± se) of the three triple *csfad2* mutants. (D) Phenotype of two‐month‐old double homozygous *csfad2* mutants compared to wild type (WT). Wild‐type *CsFAD2‐1, CsFAD2‐2* and *CsFAD2‐3* alleles are, respectively, noted 1, 2 and 3 and mutated allele (−).

Strikingly, while 5 of 19 (26%) of the T1 lines transformed with the sgRNA2 construct already showed the presence of at least one mutation among the 6 homeologous copies of *CsFAD2*, none of the 99 T1 lines transformed with sgRNA1 showed any *CsFAD2* sequence modification (Table 1). We then selected 5 mutated sgRNA2 lines and 6 randomly chosen sgRNA1 lines for amplification, with about 10 plants for each T1 line (Figure S3A). Leaf DNA from a total of 97 T2 lines was then sequenced with specific primers for the presence of mutations among the three *CsFAD2* genes. The different polymorphisms found between the three FAD2 homeologous sequences (Figure S1B) were used to develop allele‐specific primers for simple allele‐discriminating PCR (Table S1 and Figure S2). Mutations could be identified in 20/53 (37.7%) and 36/44 (81.8%) of the sgRNA1 and sgRNA2 T2 lines, respectively (Table 1). Finally, the progeny of 5 T2 sgRNA2 lines was amplified to give 133 individual T3 lines (Figure 1A), and their leaf DNA was individually sequenced for each *FAD2* copy (Table S2). A total of 21 different mutant alleles were identified, ranging from insertions or deletions of 1 to a few nucleotides, to larger deletions that would be predicted to impact FAD2 protein structure differently (Table 3). Several mutations had deletion of 3 or multiples of 3 nucleotides (6, 18 or 33 nucleotides), leading to the deletion of 1–11 amino acids in the protein, but most of the sequence changes observed were frameshift mutations or large deletions or insertions leading to important protein modification, often with premature stop codons (Table 3). Some mutations were found in all three *CsFAD2* homeologues, while others were specific to one or two genes (Table 2). The different combinations of alleles found in the T3 generation at the three *CsFAD2* loci are summarized in Table S2.

**Table 2:**
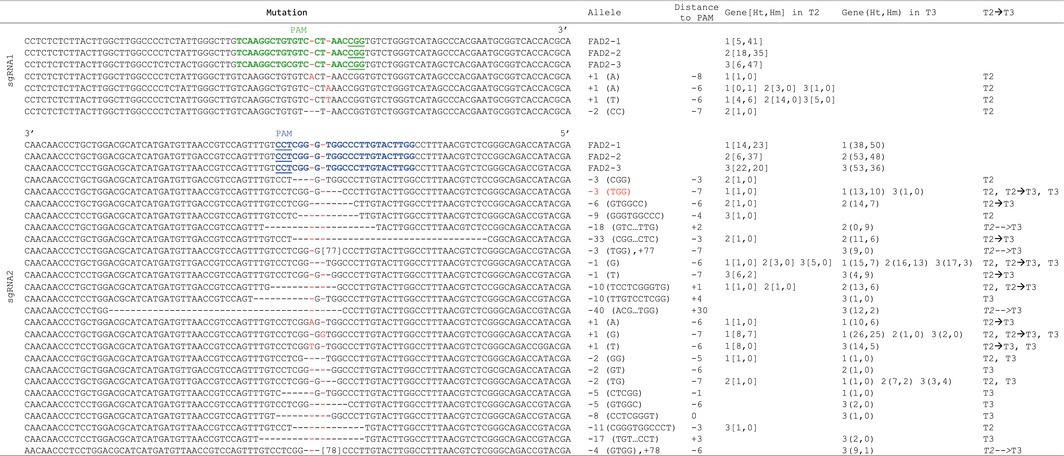
FAD2 sequences from wild type and *FAD2* CRISPR lines. The mutations in *FAD2* genes caused by each sgRNA were compared to the three FAD2 homeolog sequences. The table lists the sequence of the different *FAD2* alleles, type of mutations (+, insertion; −, deletion), the *FAD2* homeolog involved (1 for FAD2‐1, 2 for FAD2‐2 and 3 for FAD2‐3), the number of mutations in heterozygous (Ht) and homozygous (Hm) states in T2 and T3 generations, and whether the mutations were present in T2 or T3 generation (T2, T3), present in T2 and found in all the T3 lines (T2–>T3) or present in all T3 lines but not detected in T2, most probably because Cas9‐induced mutations occurred after T2 genotyping (T2‐‐>T3). The mutation leading to W194 deletion is highlighted in red

### Mutations in FAD2 genes modified the C18 desaturation profile in Camelina plants

To provide a rapid estimate of FAD2 activity in the different mutated lines, the levels of 18 carbon fatty acids were first analysed in pooled T3 seeds from single T2 progenies (Figure S3A). An oleic acid index (OAI) corresponding to the relative levels of monounsaturated oleic acid (18:1) among 18‐carbon saturated and polyunsaturated fatty acids (18:0, 18:1, 18:2 and 18:3) was used to rapidly compare the different lines for FAD2 activity. Fatty acid analysis of pooled seeds from T2 plants harbouring or not mutations in *CsFAD2* genes showed significant differences in several lines. While the wild‐type OAI varied between 14% and 18%, the progeny of several T2 lines showed an OAI between 25% and 31%, with one line reaching 41% (Figure S3A). The increase in 18:1 was mainly associated with a reduction in 18:2. Several seeds from each T2 progeny were grown, and OAI was determined from leaves of each T3 plants. Individual lipid phenotyping of T3 plants allowed rapid screening of the different lines and confirmed the extent of 18:1 increase coupled to 18:2 and 18:3 reduction (Figures 1A and S3B). As expected, while OAI in wild‐type leaves was around 8%, 96 CRISPR lines of 176 showed an OAI at least twice higher (>16%), with three lines reaching more than 35% oleic acid of the 18‐carbon fatty acid content (Figure 1A). OAI increase correlated with the number of mutated *fad2* alleles (Figure 1A). The three lines showing the highest OAI were the only plants showing drastic developmental defects, with slow growth, twisted leaves and delayed bolting and harbouring mutations in all *CsFAD2* genes (Figure 1B and C). On the contrary, the double mutants homozygous for any combinations of 2 *CsFAD2* genes did not show any developmental modifications (Figure 1D).

The other lines showed combinations of alleles with homozygous, heterozygous or biallelic combinations for the different *CsFAD2* homeologous loci (Figure 1A and Table S2). The OAI increased proportionally with the occurrence of *fad2* mutations across the 6 possible homeologous alleles, with a correlation between the number of mutated alleles and 18 : 1 levels (Figure 1A). The OAI could be the result of the number of mutated *fad2* alleles, but also the type of allele. The predicted protein sequences from the different *fad2* alleles suggested potentially weak or strong mutated alleles corresponding to deletion or insertion with or without sequence frameshift, respectively (Table 3). Of the 21 mutations recovered in T3 CRISPR populations, two alleles resulted in the deletion of single amino acid, glycine 193 or tryptophan 194 leading potentially to the weakest phenotype (Table 3). Tryptophan 194 deletion (W^194^∆) was analysed further since this residue was always included in several deletions involving 2, 3, 6 or 11 amino acids (Table 3). The different lines carrying the W^194^∆ deletion showed an OAI identical to lines carrying frameshift alleles leading to truncated proteins (Figure 1A). For instance, the lines 2F4.29 (plain arrow) and 2A6.6 (dashed arrow) carried 5 mutated *fad2* alleles. While 2F4.29 carried a –G^579^ (frameshift) at 5 of the 6 possible CsFAD2 positions, 2A6.6 showed the following deletions for, respectively, CsFAD2‐1, CsFAD2‐2 and CsFAD2‐3: TGG^580^ (W^194^∆), –GTGGCC^583^ (WP^195^∆) and a 40 bp (−23 to +17 around the PAM). The line 2F4.29 carried 5 frameshift mutations leading to potentially inactive FAD2 desaturases, while 2A6.6 carried 1 frameshift mutation for CsFAD2‐3 and 4 in frame amino acid deletion involving W194 leading to a FAD2 desaturase potentially still active. However, both lines showed very similar OAI. 2F4.29 showed a lower OAI in T4 seeds compared to 2A6.6 (52.9% and 59.8% respectively) but higher in T3 leaves (33.7% and 31%) (Table S2). The other deletion alleles involved 2 or more amino acids always including W194. These results indicated that W194 is probably essential for FAD2 activity and that oleic acid accumulation in the different lines resulted mostly from the combinatorial distribution of equivalent strong alleles rather than the effect of specific alleles.

**Table 3:**
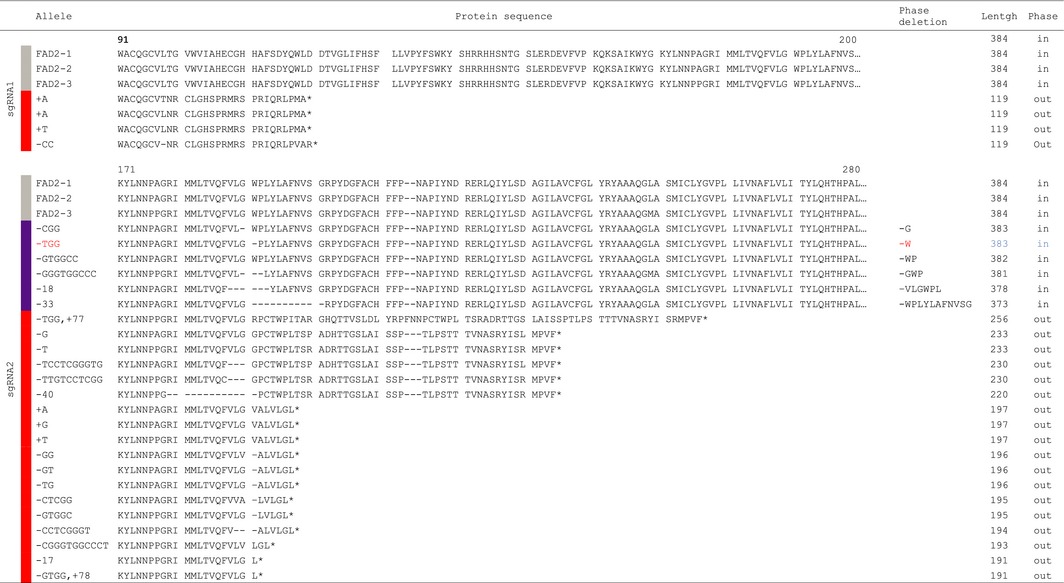
Predicted effect of sgRNA mutations on FAD2 proteins. The table shows for each allele the predicted protein sequence between residues 171 and 280; the corresponding deletion, if applicable; the final length of the protein, if the mutation led to the presence of frameshift with an out‐of‐phase sequence (out). A simple deletion of amino acids in phase with the original protein sequence is indicated by (in), and W194 is highlighted in red

### Gene dosage effect on the oleic acid profile of Camelina seed and oil

**Figure 2:**
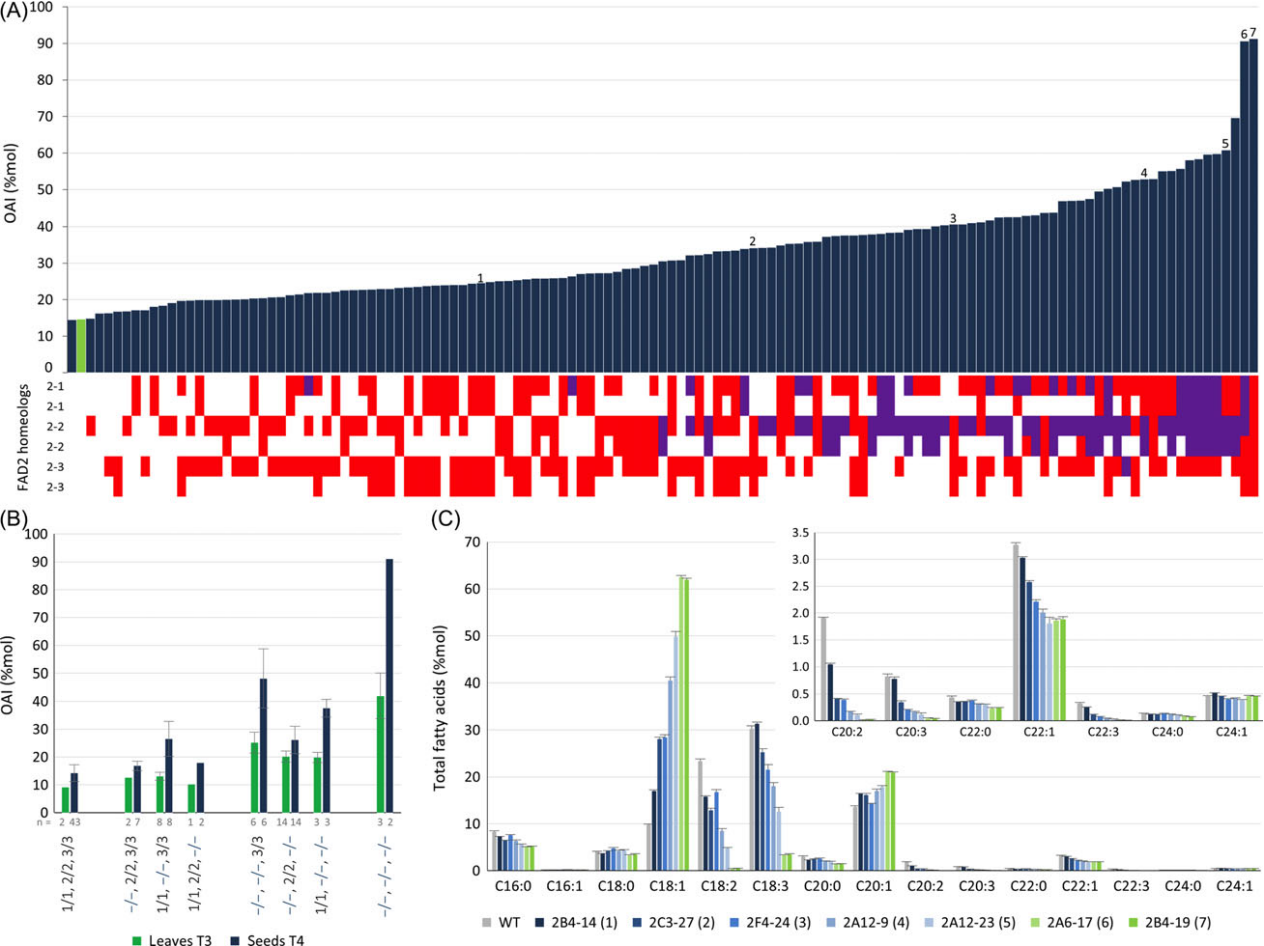
Combinatorial *csfad2* alleles changes seed lipid profile. (A) Distribution of *csfad2* allele combinations at the *CsFAD2‐1, CsFAD2‐2* and *CsFAD2‐3* loci in individual T3 lines (bottom) and the resulting effect on OAI of T4 seed progeny (top). Numbers indicate the lines selected for oil analysis (line names are indicated in C). Mutations leading to sequence frameshift are indicated in red, while those only associated with deletion or insertion without frameshift are indicated in purple. (B) OAI in simple, double and triple homozygous *csfad2* mutants. Uppercase, WT allele. Blue lowercase, mutated allele. The number of lines used for each genetic combination is indicated (n). (C) Relative fatty acid profile of T4 seeds from 7 *csfad2* CRISPR lines selected in (A).

The progeny of individual T3 lines were harvested, and the OAI of pooled T4 seeds was determined to evaluate the effect of the different combinations of *fad2* alleles on seed and oil lipid profile (Figures 2A and S3C). The T4 seeds were also scored for DsRed fluorescence, indicating the segregation of the CRISPR‐Cas9 transgene in several lines and the possibility of recovering lines without Cas9/sgRNA constructs (Table S2). As expected, the OAI observed in T4 seeds was higher than that observed in T3 leaves, with an index ranging from 14% for wild type, up to 91% for the triple mutants (Figure 2A). It should be noted that only two of the three triple homozygous *fad2* lines (2A6‐17 and 2B4‐19) were included in the analysis, since the third line yielded almost no seeds (2B4‐9). The oleic content was proportional to the number of mutated *CsFAD2* alleles, and a combination of 5–6 mutated alleles was required for achieving the highest OAI (Figures 2B and S3C and S4). Interestingly, the combination of *fad2* alleles associated with the highest OAI in T4 seeds was different from those observed in T3 leaves (Figures 1A and 2A). In seeds, the presence of homozygous *csfad2‐2* mutations was always associated with high OAI in double‐mutant combinations, while in leaves, mutations in the three *CsFAD2* genes contributed equally to oleic acid accumulation (Figure 2B). The effect of *csfad2‐2* mutation on OAI could even be observed in the heterozygous state, indicating the importance of allele dosage for FAD2 activity (Figure S4). It was reported that Camelina subgenome 3 (CsG3) was globally more expressed than the two other subgenomes (Kagale *et al*., 2016). CsFAD2‐2 belongs to CsG3, and indeed, its expression is higher in maturing seeds compared to the two other homeologous genes (Figure S5). Expression levels of the three CsFAD2 genes were similar in leaves, explaining the equal importance of the different mutated CsFAD2 alleles in leaf OAI (Figures 2B and S4).

We then selected 7 CRISPR lines with a seed OAI ranging from 8% (wild‐type Céline) to 91% (triple mutant) to determine the total fatty acid profile in seeds (Figure 2A, lines numbered 1–7). These lines corresponded to 5 double and 2 triple mutants with the following genetic backgrounds: #1: −/−, 2/2, −/−; #2: 1/1, −/−, −/−; #3: 1/1, −/−, −/−; #4: −/−, −/−, 3/3; #5: −/−, −/−, 3/3; #6: −/−, −/−, −/− and #7: −/−, −/−, −/−. Total oleic acid content ranged from 9.8% (Céline) to 62.5% (2B4‐19, line #7) and was associated with a slight increase in eicosenoic acid (20:1), but a decrease in erucic acid (22:1) (Figure 2C). As expected, polyunsaturated fatty acid levels of 18:2 and 18:3, but also of 20:2, 20:3 and 22:3, were reduced to the same extent as the increase in oleic acid (Figure 2C). Interestingly, saturated fatty acids such as 16:0, 20:0, 22:0 and 24:0 showed also a slight decrease proportional to the increase in oleic acid. Total fatty acid content in seeds varied among the different lines, but was not correlated with the relative levels of oleic acid (Figure S6A). Finally, the seeds were mechanically pressed to extract oil. The oil fraction and the residual seed cake showed a similar profile compared to seeds, with a maximum of 61.4% oil oleic acid (Figures S6B and S6C).

## Discussion

Selective mutagenesis of *CsFAD2* genes by CRISPR‐Cas9 was highly effective in Camelina. We showed efficient mutant recovery at the three *CsFAD2* genes, allowing a large diversity of genetic combinations with single, double and triple mutants that were obtained in only 3 generations (representing a total of 1 year for Camelina). Even with a large collection of natural accessions representing the different alleles used in this study, classical breeding could not have achieved the same result so quickly and so easily. Our results showed that mutagenesis was variable between generations with mutations detected for instance in leaves and not present in the seed progeny indicating the existence of somatic mutations. Mutations taking place later in development are most likely to be transmitted to the next generation if they occur in germinal tissues. Cases where mutation could be detected in T2 but not in T1 have already been reported in Arabidopsis in previous studies (see, e.g. Feng *et al*., 2014) especially when using specific promoters (Mao *et al*., 2016). Two hypotheses could be proposed to explain the fact that mutations were not detected in T1 generation using sgRNA#1. First, the absence of detected mutation in T1 could be due to a weak activity of this particular sgRNA compared to sgRNA#2. In this case due to the limit of detection of mutations in chimeric leaf tissues, we would not be able to detect the rare events of somatic mutations due to sgRNA#1. Mutagenesis efficiency was indeed variable, according to the nature of the sgRNA and time (number of generations) during which CRISPR‐Cas9 was present. Mutagenesis efficiency varied between 0% and 26% in T1 for the two sgRNA, 37% and 81.8% in T2 and reached 98.4% in T3 for sgRNA2. The higher efficiency of the sgRNA2 compared to sgRNA1 could be explained by the difference in the targeted sequence. Nucleotide composition of the sgRNA can influence the efficiency of mutagenesis as showed by the CRISPOR program that evaluates the guide activity using prediction algorithms against seven available data sets (Haeussler *et al*., 2016). sgRNA2 could be considered more efficient than sgRNA1 in 5 of the 7 algorithms used. Moreover, sgRNA2 showed 5 G or C in the final 6 bp of the sgRNA that has been shown to facilitate cleavage (Ren *et al*., 2014). Another possibility could be a specific profile of expression for the sgRNA#1, which is under the control of a Camelina U3 promoter (sgRNA#2 is under the control of a Camelina U6 promoter). In this case, the sgRNA#1 would be expressed more predominantly in specific tissues (floral tissues for example) and would thus not be present in leaf tissue. In Arabidopsis, the AtU3 promoter had relatively weaker activity than the AtU6 (Zhang *et al*., 2016). If this feature is conserved in Camelina, one could expect that the CsU3 would also have a relatively weaker activity than the CsU6 promoter explaining the lower efficiency of sgRNA1 compared to sgRNA2.

The nature of mutations induced by the two sgRNA seems also to differ. sgRNA1 induced essentially insertions of 1 bp and one deletion, while sgRNA2 led essentially to larger range of deletions, some insertions and few substitutions. Such differences between sgRNA have already been observed in plants and can be explained by the difference in DNA repair pathway used by the cell to resolve the double‐strand break (DSB) (Collonnier *et al*., 2016). Indeed, two repair pathways exist that can resolve the DSB in the absence of a recombination matrix, the C‐NHEJ and alternative end joining (alt‐EJ) mechanisms (Sfeir and Symington, 2015). C‐NHEJ‐mediated repair can be precise or associated with small insertions or deletions at the cut site. Alt‐EJ repair involves alignment of microhomologous sequences internal to the broken ends before joining and is associated with deletions flanking the original DSB. Thus, the difference in the type of mutations that we observe between sgRNA1 and sgRNA2 could be explained by the respective abundance of microhomologies in the vicinity of the DSB generated by the two sgRNAs. Concerning sgRNA2, a large number of the observed deletions could be explained by alt‐EJ repair (e.g. ‐T in position 15 possibly due to the GG repeats; ‐33 in position 11 possibly due to GTC repeats). For sgRNA1, the possibility of microhomology‐mediated repair seems more limited and the DSB is probably more resolved through C‐NHEJ. This could explain why only single A or T insertions are observed with sgRNA1. Moreover, because alt‐EJ is considered more mutagenic than C‐NHEJ, this differential content of microhomologies between the two sgRNAs could also participate to the higher mutagenic capacity of sgRNA2. The analysis of larger number of sgRNA‐mediated mutations is however necessary to confirm these hypotheses.

In Arabidopsis that was transformed using a method similar to that used for Camelina, the extensive analysis of several hundred lines across 12 different target genes demonstrated that the mutation rate in T1 was on average 71% (47%–92%) (Feng *et al*., 2014). The mutagenesis rate observed in Camelina was in the range of multigene targeting in Arabidopsis. However, more sgRNA should be tested to evaluate thoroughly the efficiency of this technique in Camelina. Since efficiency increased rapidly with generation time, selfing and analysis of the next generation provides a simple and efficient way to achieve mutant identification and recovery. CRISPR‐Cas9 was also used in hexaploid wheat to inactivate the three homeologous Ta‐MLO genes, conferring resistance to powdery mildew (Wang *et al*., 2014). Contrary to Arabidopsis and Camelina, protoplast transformation was used, and among the 72 transgenic wheat plants regenerated, only 4 (5.6%) carried mutations in one Ta‐MLO‐A1 homeolog. In the same study, particle bombardment of immature embryos with a TALEN construct led to a similar mutagenesis efficiency (6%). However, the larger population of plants screened with the TALEN constructs allowed identification of mutations in the three homeologous Ta‐MLO genes. In conclusion, flower dip Agrobacterium‐mediated transformation of CRISPR/Cas9 of Camelina could provide an efficient way to mutagenize polyploid crop species.

Unlike wheat, Camelina has three similar subgenomes that are generally expressed, indicating the existence of an important functional gene redundancy (Kagale *et al*., 2014, 2016). Our work shows that CRISPR‐Cas9 could efficiently bypass this limitation by quickly isolating triple homozygous mutants. The other advantage of CRISPR/Cas9 in a polyploid plant that has three similar subgenomes is the possibility of creation of a large collection of combinatorial alleles across the different homeolog genes. This large and specific mutant diversity could then be used to genetically fine‐tune the expected phenotype by selecting the appropriate allelic combination. In particular, fatty acid desaturation appeared to be very sensitive to gene dosage, and one could even detect the effect of a single heterozygous *fad2* mutation among the mutated population. A large collection of mutants provides tools to investigate the quantitative involvement of the different homeologous genes in the associated phenotype. Combinatorial distribution of alleles across the three CsFAD2 homeologs for a given oleic acid profile was different in leaves and seeds indicating a different contribution of *CsFAD2* genes in different organs. In particular, *CsFAD2‐2* was found to have a more important contribution in oleic acid desaturation in seeds compared to *CsFAD2‐1* and *CsFAD2‐3* in double‐mutant combinations, while the three genes seem to have a similar contribution in leaves. This genetic diversity could be further extended by looking for mutations in different regions of the genes or by selecting known weak alleles. We recovered mostly null mutations with our sgRNAs, but we could have obtained weaker mutations by targeting sgRNA at the 3′ end of the gene or introducing specific mutations by CRISPR‐Cas9‐induced gene recombination.

Among the population of mutated *fad2* lines, the three triple homozygous lines showed a pronounced developmental phenotype. High oleic Camelina lines have already been obtained with seed‐specific *FAD2* antisense or RNAi suppression, leading to 50% 18:1 in seeds (Kang *et al*., 2011; Nguyen *et al*., 2013). Simultaneous inactivation of *fad2* and *fae1* by RNAi in the seed could increase 18:1 accumulation to 70% of total seed fatty acids, but also without any somatic phenotype (Nguyen *et al*., 2013). A *fad2* Camelina mutant was isolated from an EMS‐mutagenized Camelina population, but since only one *FAD2* homeolog was inactivated, 18:1 levels reached only 27% of total seed fatty acids (Kang *et al*., 2011). In Arabidopsis, several *fad2*‐deficient lines have been isolated either by chemical mutagenesis (James and Dooner, 1990, 1991; Lemieux *et al*., 1990; Song *et al*., 2010), T‐DNA insertions (Okuley *et al*., 1994; Zhang *et al*., 2012) or hpRNA (Stoutjesdijk *et al*., 2002). Most of the lines showed oleic acid levels around 20%–26% in leaves and 50%–57% in seeds, and did not show strong phenotypes when grown under normal conditions (Lemieux *et al*., 1990; Miquel *et al*., 1993; Okuley *et al*., 1994; Song *et al*., 2010; Stoutjesdijk *et al*., 2002; Zhang *et al*., 2012). The fact that Camelina complete inactivation of FAD2 was associated with a strong phenotype raised the question of the role of polyunsaturated fatty acids in plant development. An unlikely explanation is the presence of unrelated off‐target mutations induced by CRISPR/Cas9 since several independent lines were selected. Another possibility is that the highest levels of polyunsaturated fatty acid in Camelina membranes compared to Arabidopsis enhance its sensitivity to environmental conditions. Indeed, Arabidopsis *fad2* mutants were associated with cold and salt sensitivity (Miquel *et al*., 1993; Zhang *et al*., 2012). A stunted bushy phenotype was nonetheless observed in one of the most extreme *fad2* mutants that accumulated 66.4% oleic acid in seeds, and the phenotype was amplified in the double mutant *fae1fad2* which accumulated 86.9% 18:1 (James and Dooner, 1990, 1991), indicating that even in Arabidopsis, polyunsaturated fatty acids are important for plant growth.

In conclusion, CRISPR‐Cas9 generates a large targeted genetic diversity that helps in understanding gene function in polyploid species, but also provide an exceptional genetic resource for breeding (Nogué *et al*., 2016). In particular, it provides a very efficient method for selecting an ideotype (oil profile), minimizing negative effects (growth trade‐off), by selecting the nature of the alleles and their most efficient genetic combinations. In the case of ancient crops that have undergone little breeding improvement such as Camelina, CRISPR‐Cas9 genome editing provides major genetic leverage to improve its growing agronomical and biotechnological potential (Faure and Tepfer, 2016).

## Material and methods

### sgRNA synthesis and cloning

Two different guide RNAs were designed following the guide RNA architectures (20nt‐NGG). The research of the guide RNA was based on the genome sequence of Arabidopsis using the TEFOR website (http://crispor.tefor.net). The two sgRNA sequences were eventually selected for their consensus sequence with the three Camelina *FAD2* genes (Figure S1). The genomic sequence of *Camelina sativa* cv. Céline Cs*FAD2‐1, CsFAD2‐2* and *CsFAD2‐3* was named according to the previous work (Kang *et al*., 2011) and was respectively related to the accessions *Csa19 g016350, Csa01 g013220* and *Csa15 g016000* (Kagale *et al*., 2016) and *CsFAD2A, CsFAD2B* and *CsFAD2C* (Hutcheon *et al*., 2010).

Specificity of the sgRNA for the *FAD2* genes was finally checked by BLAST search of the two‐guide RNA against Camelina genome in NCBI. The guide RNA 1 (5′**a**tgtcaaggctgtgtcctaac3′) and guide RNA 2 (5′**g**ccaagtacaagggccacccg3′) were synthesized, respectively, with Camelina U3 and Camelina U6 promoter sequences and flanked by Gateway (Thermofisher Lifetech) attb1 and attb2 recombination sites. Camelina U3 and Camelina U6 promoters used in this study (Figure S7) were identified by Basic Local Alignment Search Tool (http://www.phytozome.net/physcomitrella_er.php) using the Arabidopsis U6‐26 and U3B snRNA sequences (X52528 and X52629 respectively) as queries.

Those cassettes were synthesized by GenScript and cloned individually into pDONR207 (Life Technologies) by Gateway recombination, resulting in a set of pDONR207‐sgRNA vectors in which the guide RNA cassettes could be later recombined into pDE‐Cas9 (Fauser *et al*., 2014) using the Gateway LR clonase. The pDE‐Cas9 vector (kind gift from Holger Puchta, Karlsruhe Institute of Technology) was modified by exchanging the Basta resistance cassette (*SpeI* and *PmeI*) with a DsRed cassette (*AvrII* and *HpaI*).

### Camelina transformation and transformant selection

Camelina Celine (cultivar) was transformed following an improved method of the traditional Arabidopsis floral‐dip method (Clough and Bent, 1998). Flower of Camelina were dipped in a solution of agrobacterium supplemented with sucrose (73 mm), MgCl_2_ (12.6 mm) and acetosyringone (100 μm) in a vacuum under low pressure (50 mbar). Transgenic lines were selected by the detection of the DsRed marker fluorescence in seeds (Julié‐Galau *et al*., 2014).

### Mutation detection and analysis

DNA was extracted from Camelina leaves using the DNeasy^®^ Plant Mini Kit (Qiagen) according to the manufacturer's instructions. The presence of mutations was first detected by sequencing PCR amplicons using consensus primers for the three *FAD2* genes (CDSFAD2_For145‐CsFAD2‐FN‐Rev). To determine the nature (allele sequence, *FAD2* gene), each *FAD2* gene was amplified at either sgRNA1 or sgRNA2 sites by the simple allele‐discriminating PCR (SAP) (Biu and Liu, 2016). At least one specific primer in forward (forward FAD2‐2: CsFad2b_SNP_F; forward FAD2‐3: CsFad2c_SNP_F) or reverse direction (reverse FAD2‐1: CsFad2a_SNP_R2; reverse FAD2‐2: CsFad2b_SNP_R; reverse FAD2‐3: CsFad2c_SNP_R) was used with a reverse (CsFAD2_Enz_Rev) or forward (CsFAD2_Enz_For) primers common to the three copies. Sequencing was carried out with CDSFAD2_For145/CsFAD2_553_Rev primers for ARNg1 and CsFAD2_396_For/CDSFAD2_Rev812 primers for ARNg2. All primers used are described in Table S1 and Figure S2. Mixed Sequences Reader program (http://msr.cs.nthu.edu.tw/) was used to analyse mixed sequence caused by heterozygous mutations.

### Lipid analysis

Camelina leaves were immediately frozen after sampling at −80 °C and then lyophilized. For the quantification of the fatty acid methyl esters (FAMEs), around 2 mg of dry leaves or 20 seeds were used, and FAMEs extraction followed by GC‐MS analysis was performed according to Li *et al*. (2006). The ratio of 18 : 1/(18 : 0 + 18 : 1 + 18 : 2 + 18 : 3)*100 was calculated to define the oleic acid index (OAI). Oil was extracted by a home‐made centrifugation‐based micropress device that can efficiently separate oil fraction from residual seed cake (Faure and Tepfer, 2016).

## Supporting information


**Figure S1.** Structure of Camelina and Arabidopsis *FAD2* genes.
**Figure S2.** Camelina *CsFAD2* coding sequences and the different primers used in the study.
**Figure S3.** Combinatorial *fad2* alleles associated with C18 content in T2 and T3 *CsFAD2* CRISPR lines.
**Figure S4.** OAI for the different allelic combinations at the three *CsFAD2* loci.
**Figure S5.** Expression levels of *CsFAD2* genes.
**Figure S6.** Fatty acid content of oil and cake fractions of selected *CsFAD2* CRISPR lines analyzed in Figure 2A and C.
**Figure S7.** Camelina U3 and U6 promoters used in this study.
**Table S1.** Primer sequences used for amplification and sequencing.

**Table S2.** Summary of the different genotypes of *CsFAD2* CRISPR T2 and T3 lines.
