# Supplementary material for: Selective gene dosage by CRISPR‐Cas9 genome editing in hexaploid Camelina sativa
Source: Plant Biotechnol J. 2017 Apr 1;15(6):729–39. doi: 10.1111/pbi.12671 (PMC5425392; doi:10.1111/pbi.12671)
Supplement: Supplementary file 1 — Figure S1. Structure of Camelina and Arabidopsis FAD2 genes. Figure S2. Camelina CsFAD2 coding sequences and the different primers used in the study. Figure S3. Combinatorial fad2 alleles associated with C18 content in T2 and T3 CsFAD2 CRISPR lines. Figure S4. OAI for the different allelic combinations at the three CsFAD2 loci. Figure S5. Expression levels of CsFAD2 genes. Figure S6. Fatty acid content of oil and cake fractions of selected CsFAD2 CRISPR lines analyzed in Figure 2A and C. Figure S7. Camelina U3 and U6 promoters used in this study. Table S1. Primer sequences used for amplification and sequencing. [file PBI-15-729-s001.pdf]

A

```
ATG GGT GCA GGT GGA AGA ATG CCG GTT CCT ACT TCT TCC AAG AAA TCG GAA ACC GAC ACC ACA AAG CGT GTG CCG TGC GAG AAA CCG CCT TTC TCG GTG GGA GAT CTG AAG AAA GCA ATC
M   G   A   G   G   R   M   P   V   P   T   S   S   K   K   S   E   T   D   T   T   K   R   V   P   C   E   K   P   P   F   S   V   G   D   L   K   K   A   I
1/1                 31/11               61/21               91/31
CCG CCG CAT TGT TTC AAA CGC TCA ATC CCT CGC TCT TTC TCC TAC CTT ATC AGT GAC ATC ATT ATA GCC TCA TGC TTC TAC TAC GTC GCC ACC AAT TAC TTC TCT CTC CTC CCT CAG CCT
P   P   H   C   F   K   R   S   I   P   R   S   F   S   Y   L   I   S   D   I   I   I   A   S   C   F   Y   Y   V   A   T   N   Y   F   S   L   L   P   Q   P
121/41                                  151/51                          181/61                                  211/71
CTC TCT TAC TTG GCT TGG CCA CTC TAT TGG GCC TGT CAA GGC TGT GTC CTA ACT GGT ATC TGG GTC ATA GCC CAC GAA TGC GGT CAC CAC GCA TTC AGC GAC TAC CAA TGG CTG GAT GAC
L   S   Y   L   A   W   P   L   Y   W   A   C   Q   G   C   V   L   T   G   I   W   V   I   A   H   E   C   G   H   H   A   F   S   D   Y   Q   W   L   D   D
241/81                          271/91                          301/101                         331/111
ACA GTT GGT CTT ATC TTC CAT TCC TTC CTC CTC GTC CCT TAC TTC TCC TGG AAG TAT AGT CAT CGC CGT CAC CAT TCC AAC ACT GGA TCC CTC GAA AGA GAT GAA GTA TTT GTC CCA AAG
T   V   G   L   I   F   H   S   F   L   L   V   P   Y   F   S   W   K   Y   S   H   R   R   H   H   S   N   T   G   S   L   E   R   D   E   V   F   V   P   K
361/121                                 391/131                         421/141                                 451/151

CAG AAA TCA GCA ATC AAG TGG TAC GGG AAA TAC CTC AAC AAC CCT CTT GGA CGC ATC ATG ATG TTA ACC GTC CAG TTT GTC CTC GGG TGG CCC TTG TAC TTA GCC TTT AAC GTC TCT GGC
Q   K   S   A   I   K   W   Y   G   K   Y   L   N   N   P   L   G   R   I   M   M   L   T   V   Q   F   V   L   G   W   P   L   Y   L   A   F   N   V   S   G
481/161                                 511/171                         541/181                 571/191

AGA CCG TAT GAC GGG TTC GCT TGC CAT TTC TTC CCC AAC GCT CCC ATC TAC AAT GAC CGA GAA CGC CTC CAG ATA TAC CTC TCT GAT GCG GGT ATT CTA GCC GTC TGT TTT GGT CTT TAC
R   P   Y   D   G   F   A   C   H   F   F   P   N   A   P   I   Y   N   D   R   E   R   L   Q   I   Y   L   S   D   A   G   I   L   A   V   C   F   G   L   Y
601/201                         631/211                         661/221                         691/231

CGT TAC GCT GCT GCA CAA GGG ATG GCC TCG ATG ATC TGC CTC TAC GGA GTA CCG CTT CTG ATA GTG AAT GCG TTC CTC GTC TTG ATC ACT TAC TTG CAG CAC ACT CAT CCC TCG TTG CCT
R   Y   A   A   A   Q   G   M   A   S   M   I   C   L   Y   G   V   P   L   L   I   V   N   A   F   L   V   L   I   T   Y   L   Q   H   T   H   P   S   L   P
721/241                                 751/251                         781/261                         811/271
CAC TAC GAT TCA TCA GAG TGG GAC TGG CTC AGG GGA GCT TTG GCT ACC GTA GAC AGA GAC TAC GGA ATC TTG AAC AAG GTG TTC CAC AAC ATT ACA GAC ACA CAC GTG GCT CAT CAC CTG
H   Y   D   S   S   E   W   D   W   L   R   G   A   L   A   T   V   D   R   D   Y   G   I   L   N   K   V   F   H   N   I   T   D   T   H   V   A   H   H   L
841/281                                 871/291                 901/301                         931/311
TTC TCG ACA ATG CCG CAT TAT AAC GCA ATG GAA GCT ACA AAG GCG ATA AAG CCA ATT CTG GGA GAC TAT TAC CAG TTC GAT GGA ACA CCG TGG TAT GTA GCG ATG TAT AGG GAG GCA AAG
F   S   T   M   P   H   Y   N   A   M   E   A   T   K   A   I   K   P   I   L   G   D   Y   Y   Q   F   D   G   T   P   W   Y   V   A   M   Y   R   E   A   K
961/321                         991/331                         1021/341                        1051/351

GAG TGT ATC TAT GTA GAA CCG GAC AGG GAA GGT GAC AAG AAA GGT GTG TAC TGG TAC AAC AAT AAG TTA TGA
E   C   I   Y   V   E   P   D   R   E   G   D   K   K   G   V   Y   W   Y   N   N   K   L   *
1081/361                                1111/371                        1141/381
```

B

```
AtFAD2   ATG  ...  G CCT CTC TCT TAC TTG GCT TGG CCA CTC TAT TGG GCC TGT CAA GGC TGT GTC CTA ACT GGT ATC TGG GTC ATA GCC CAC GAA TGC GGT CAC C
                    P   L   S   Y   L   A   W   P   L   Y   W   A   C   Q   G   C   V   L   T   G   I   W   V   I   A   H   E   C   G   H   H

CsFAD2-1 ATG  ...  G CCT CTC TCT TAC TTG GCT TGG CCC CTC TAT TGG GCT TGT CAA GGC TGT GTC CTA ACC GGT GTC TGG GTC ATA GCC CAC GAA TGC GGT CAC C
CsFAD2-2 ATG  ...  G CCT CTC TCT TAC TTG GCT TGG CCC CTC TAT TGG GCT TGT CAA GGC TGT GTC CTA ACC GGT GTC TGG GTC ATA GCC CAC GAA TGC GGT CAC C
CsFAD2-3 ATG  ...  G CCT CTC TCT TAC TTG GCT TGG CCT CTC TAC TGG GCT TGT CAA GGC TGC GTC CTA ACC GGT GTC TGG GTC ATA GCT CAC GAA TGC GGT CAC C
         M          P   L   S   Y   L   A   W   P   L   Y   W   A   C   Q   G   C   V   L   T   G   V   W   V   I   A   H   E   C   G   H   H
         1         240        250         260         270         280         290         302         310         320         330


AtFAD2   AC GCA TTC AGC GAC TAC CAA TGG CTG GAT GAC ACA GTT GGT CTT ATC TTC CAT TCC TTC CTC CTC GTC CCT TAC TTC TCC TGG AAG TAT AGT CAT CGC CG
            A   F   S   D   Y   Q   W   L   D   D   T   V   G   L   I   F   H   S   F   L   L   V   P   Y   F   S   W   K   Y   S   H   R   R

CsFAD2-1 AC GCA TTC AGC GAC TAC CAA TGG CTT GAT GAC ACA GTT GGT CTT ATC TTC CAT TCC TTC CTT CTC GTC CCT TAC TTC TCC TGG AAG TAC AGT CAT CGC CG
CsFAD2-2 AC GCA TTC AGC GAC TAC CAG TGG CTC GAT GAC ACA GTC GGT CTT ATC TTC CAT TCC TTC CTT CTC GTC CCT TAC TTC TCC TGG AAG TAC AGT CAT CGC CG
CsFAD2-3 AC GCA TTC AGC GAC TAC CAA TGG CTT GAT GAC ACA GTT GGT CTT ATC TTC CAT TCC TTC CTT CTC GTC CCT TAC TTC TCC TGG AAG TAC AGT CAT CGC CG
            A   F   S   D   Y   Q   W   L   D   D   T   V   G   L   I   F   H   S   F   L   L   V   P   Y   F   S   W   K   Y   S   H   R   R
               340         350         360         370         380         390         400         410         400         430


AtFAD2   T CAC CAT TCC AAC ACT GGA TCC CTC GAA AGA GAT GAA GTA TTT GTC CCA AAG CAG AAA TCA GCA ATC AAG TGG TAC GGG AAA TAC CTC AAC AAC CCT CTT
            H   H   S   N   T   G   S   L   E   R   D   E   V   F   V   P   K   Q   K   S   A   I   K   W   Y   G   K   Y   L   N   N   P   L

CsFAD2-1 T CAC CAT TCC AAC ACA GGA TCT CTC GAA AGA GAT GAA GTA TTT GTC CCA AAG CAG AAG TCC GCT ATC AAG TGG TAT GGC AAA TAC CTC AAC AAC CCT GCT
CsFAD2-2 T CAC CAT TCC AAC ACA GGA TCC CTC GAA AGA GAT GAA GTA TTT GTC CCA AAG CAG AAG TCC GCT ATC AAG TGG TAT GGC AAA TAC CTC AAC AAC CCT GCT
CsFAD2-3 T CAC CAT TCC AAC ACA GGA TCT CTC GAA AGA GAT GAA GTA TTT GTC CCA AAG CAG AAA TCA GCT ATC AAG TGG TAT GGC AAA TAC CTC AAC AAC CCT CCT
            H   H   S   N   T   G   S   L   E   R   D   E   V   F   V   P   K   Q   K   S   A   I   K   W   Y   G   K   Y   L   N   N   P   A
               440         450         460         470         480         490         500         510         500         530


AtFAD2   GGA CGC ATC ATG ATG TTA ACC GTC CAG TTT GTC CTC GGG TGG CCC TTG TAC TTA GCC TTT AAC GTC TCT GGC AGA CCG TAT GAC GGG TTC GCT  ...  TGA
          G   R   I   M   M   L   T   V   Q   F   V   L   G   W   P   L   Y   L   A   F   N   V   S   G   R   P   Y   D   G   F   A

CsFAD2-1 GGA CGC ATC ATG ATG TTA ACC GTC CAG TTT GTC CTC GGG TGG CCC TTG TAC TTG GCC TTT AAC GTC TCG GGC AGA CCA TAC GAT GGG TTC GCT  ...  TGA
CsFAD2-2 GGA CGC ATC ATG ATG TTG ACC GTC CAG TTT GTC CTC GGG TGG CCC TTG TAC TTG GCC TTT AAC GTC TCC GGC AGA CCA TAC GAC GGG TTC GCT  ...  TGA
CsFAD2-3 GGA CGC ATC ATG ATG TTA ACC GTC CAG TTT GTC CTC GGG TGG CCC TTG TAC TTG GCC TTT AAC GTC TCG GGC AGA CCG TAC GAC GGG TTC GCT  ...  TGA
          G   R   I   M   M   L   T   V   Q   F   V   L   G   W   P   L   Y   L   A   F   N   V   S   G   R   P   Y   D   G   F   A       stop
               540         550         560         570         580         590         600         610         620         1155
```

C

sgRNA#1
ggggacaagtttgtacaaaaaagcaggcttcATTCAACAAGAAAAGAATATGTGTAACTGACGAAAGTTCTTAGGCTAACAAAAATGTATTATAGTGAACATAGAAATAACAGA
ATGCTTTTTTACTCGTAAACACATGATCACAAAATTCACAATCAAAGTCGGTTTAGATGTACCGTCCGGTTTAAAGCAAATAAGTTGGGAATGGGTTTTACAAATAA
ACAAGTGGGCTTTGGCCCGTATCACCTACTATTAAAGGGAACTCAACTCACATTACAATTTACAAGAGTACAAACATCCCACATCGCTCGCCTAGGAAACATAGCTGT
TGTATATATAACGTTGAGAGAGCAACGTTGGTCatgtcaaggctgtgtcctaacGTTTTAGAGCTAGAAATAGCAAGTTAAAATAAGGCTAGTCCGTTATCAACTTGAAAAAG
TGGCACCGAGTCGGTGCTTTTTTTGTCgacccagctttcttgtacaaagtggtcccc

sgRNA#2
ggggacaagtttgtacaaaaaagcaggcttcCTCGAGGTTTAAGTTATAATTCCCCCATAGGTTCTCGCTAAATCAGATACCAGTAACATTTAGCAACATCCCATAGTCTTTTTTT
TTTTTAATCAAACTTTAAACCACAAGTCATTAGCTTTAACAAATTTAAAGCTAACGTTACAAAACCAACGGCGTCGTTTCACTCAATCTCTAAGAATAATAATCAGAA
AGAGGACTTTTAATTACCTTAAATAGGCCTTTTATCAAACGAGCCCTTTTAAGCTTTAACCGATAACAAATATACCACATCGCATAGAACAGAGGACAAAAGCTGCGT
TTAAATAATGTTAGAGTCGAGTAAGTGATTgccaagtacaagggccacccgGTTTTAGAGCTAGAAATAGCAAGTTAAAATAAGGCTAGTCCGTTATCAACTTGAAAAAGTG
GCACCGAGTCGGTGCTTTTTTTGTCgacccagctttcttgtacaaagtggtcccc

FIGURE S1

```
                      *         *         *         *         *         *         *         *         *         *
CsFAD2-1   1>ATGGGTGCAGGTGGAAGAATGCCGGTTCCTTCTTCTTCTTCCAAGAAATCAGAAACCGATGCCATAAAGCGTGTGCCTTGCGAGAAACCGCCGTTCACAC>100
CsFAD2-2   1>ATGGGTGCAGGTGGAAGAATGCCAGTTCCTTCTTCTTCTTCCAAGAAATCTGAAACCGATGCCATAAAGCGTGTGCCCTGCGAGAAACCACCGTTCACGC>100
CsFAD2-3   1>ATGGGTGCAGGTGGAAGAATGCCAGTTCCTTCTTCTTCTTCCAAGAAATCTGAAACCGATGCCATAAAGCGTGTGCCCTGCGAGAAACCGCCGTTCACGC>100
                                                                  AACCGATGCCATAAAGCGTG
                                                                      CsFAD2_Enz_For →

                       *         *         *         *         *         *         *         *         *
CsFAD2-1 101>TGGGAGAAT-TGAAGAAAGCGATCCCACCGCAGTGTTTCAAACGCTCTATCCCTCGCTCTTTCTCCTACCTTATCACTGACATCATTGTTGCCTCCTGCT>199
CsFAD2-2 101>TGGGAG-ATCTGAAGAAAGCAATCCCACCGCAGTGTTTCAAACGCTCTATCCCTCGCTCTTTCTCCTACCTTATCACTGACATCATTATTGCCTCCTGCT>199
CsFAD2-3 101>TTGGAGAA-CTGAAGAAAGCAATCCCCCCGCAGTGTTTCAAACGCTCTATCCCTCGCTCTTTCTCCTACCTTATCACTGACATCATTGTTGCCTCCTGCT>199
                                                             ATCCCTCGCTCTTTCTCCTAC
                                                                 CDSFAD2_For145 →

             *         *         *         *         *         *         *         *         *         *
CsFAD2-1 200>TCTACTACGTCGCCACCAATTACTTCTCTCTCCTCCCTCAGCCTCTCTCTTACTTGGCTTGGCCCCTCTATTGGGCTTGTCAAGGCTGTGTCCTAACCGG>299
CsFAD2-2 200>TCTACTACGTCGCCACCAATTACTTTTCTCTCCTCCCTCAGCCTCTCTCTTACTTGGCTTGGCCCCTCTATTGGGCTTGTCAAGGCTGTGTCCTAACCGG>299
CsFAD2-3 200>TCTACTACGTCGCCACCAATTACTTCTCTCTCCTCCCTCAGCCTCTCTCTTACTTGGCTTGGCCTCTCTACTGGGCTTGTCAAGGCTGCGTCCTAACCGG>299

             *         *         *         *         *         *         *         *         *         *
CsFAD2-1 300>TGTCTGGGTCATAGCCCACGAATGCGGTCACCACGCATTCAGCGACTACCAATGGCTTGATGACACAGTTGGTCTTATCTTCCATTCCTTCCTTCTCGTC>399
CsFAD2-2 300>TGTCTGGGTCATAGCCCACGAATGCGGTCACCACGCATTCAGCGACTACCAGTGGCTCGATGACACAGTCGGTCTTATCTTCCATTCCTTCCTTCTCGTC>399
CsFAD2-3 300>TGTCTGGGTCATAGCTCACGAATGCGGTCACCACGCATTCAGCGACTACCAATGGCTTGATGACACAGTTGGTCTTATCTTCCATTCCTTCCTTCTCGTC>399
                                                                                                             CGTC

             *         *         *         *         *         *         *         *         *         *
CsFAD2-1 400>CCTTACTTCTCCTGGAAGTACAGTCATCGCCGTCACCATTCCAACACAGGATCTCTCGAAAGAGATGAAGTATTTGTCCCAAAGCAGAAGTCCGCTATCA>499
CsFAD2-2 400>CCTTACTTCTCCTGGAAGTACAGTCATCGCCGTCACCATTCCAACACAGGATCCCTCGAAAGAGATGAAGTATTTGTCCCAAAGCAGAAGTCCGCTATCA>499
CsFAD2-3 400>CCTTACTTCTCCTGGAAGTACAGTCATCGCCGTCACCATTCCAACACAGGATCTCTCGAAAGAGATGAAGTATTTGTCCCAAAGCAGAAATCAGCTATCA>499
             CCTTACTTCTCCTGG                                                         TTGTCCCAAAGCAGAAATGA
             CsFAD2_396_For →                                                            CsFad2c_SNP_F →

             *         *         *         *         *         *         *         *         *         *
CsFAD2-1 500>AGTGGTATGGCAAATACCTCAACAACCCTGCTGGACGCATCATGATGTTAACCGTCCAGTTTGTCCTCGGGTGGCCCTTGTACTTGGCCTTTAACGTCTC>599
CsFAD2-2 500>AGTGGTATGGCAAATACCTCAACAACCCTGCTGGACGCATCATGATGTTGACCGTCCAGTTTGTCCTCGGGTGGCCCTTGTACTTGGCCTTTAACGTCTC>599
CsFAD2-3 500>AGTGGTATGGCAAATACCTCAACAACCCTCCTGGACGCATCATGATGTTAACCGTCCAGTTTGTCCTCGGGTGGCCCTTGTACTTGGCCTTTAACGTCTC>599
                                          GCTGGACGCATCATGATGTGG     GTCCAGTTTGTCCTCGGGT
                                              CsFad2b_SNP_F →         ← CsFAD2_553_Rev

             *         *         *         *         *         *         *         *         *         *
CsFAD2-1 600>GGGCAGACCATACGATGGGTTCGCTTGCCATTTCTTCCCCAACGCTCCCATCTACAACGACCGTGAACGCCTCCAGATATATCTCTCTGATGCCGGTATT>699
CsFAD2-2 600>CGGCAGACCATACGACGGGTTCGCTTGCCATTTCTTCCCCAACGCTCCCATCTACAACGACCGTGAACGCCTCCAGATATATCTCTCTGATGCCGGTATT>699
CsFAD2-3 600>GGGCAGACCGTACGACGGGTTCGCTTGCCATTTCTTCCCCAACGCTCCCATCTACAACGACCGTGAACGCCTCCAGATATATCTCTCGGATGCCGGTATT>699
             CCGCAGACCATACGACGGGTT                CCCAACGCTCCCATCTACA
                 ← CsFad2b_SNP_R                    ← CsFAD2-FN-Rev
                      GGACGACGGGTTCGCTTG
                        ← CsFad2c_SNP_R

             *         *         *         *         *         *         *         *         *         *
CsFAD2-1 700>CTAGCAGTCTGTTTTGGGCTTTACCGTTACGCCGCTGCACAAGGATTGGCCTCGATGATCTGCCTCTACGGAGTACCACTTCTGATAGTAAACGCGTTCC>799
CsFAD2-2 700>CTAGCAGTCTGTTTTGGGCTTTACCGTTACGCCGCTGCACAAGGATTGGCCTCGATGATCTGCCTCTACGGAGTACCACTTCTGATAGTGAACGCGTTCC>799
CsFAD2-3 700>CTAGCAGTCTGTTTTGGGCTTTACCGTTACGCTGCTGCACAAGGAATGGCCTCGATGATCTGCCTCTACGGAGTACCGCTTCTGATAGTGAACGCGTTCC>799
                                                                                                      AGACGCGTTCC
                                                                                                       ← CsFad2a_SNP_R2

             *         *         *         *         *         *         *         *         *         *
CsFAD2-1 800>TCGTCTTGATCACTTACTTGCAGCACACTCATCCTGCGTTGCCTCACTACGATTCATCCGAGTGGGATTGGCTTAGGGGAGCTTTGGCTACCGTAGACAG>899
CsFAD2-2 800>TCGTCTTGATCACTTACTTGCAGCACACTCATCCTGCGTTGCCTCACTACGATTCATCCGAGTGGGATTGGCTTAGGGGAGCTTTGGCTACCGTAGACAG>899
CsFAD2-3 800>TCGTCTTGATCACTTACTTGCAGCACACTCATCCTGCGTTGCCTCACTACGATTCATCCGAGTGGGATTGGCTTAGGGGAGCTTTGGCTACCGTTGACAG>899
             TCGTCTTGA   CTTACTTGCAGCACACTCATCC
                           ← CDSFAD2_Rev812

             *         *         *         *         *         *         *         *         *         *
CsFAD2-1 900>AGACTATGGAATCTTGAACAAGGTGTTCCACAACATCACGGACACACATGTGGCTCATCATCTGTTTTCGACAATGCCGCATTATAATGCGATGGAAGCT>999
CsFAD2-2 900>AGACTATGGAATCTTGAATAAGGTGTTCCACAACATCACGGACACACATGTGGCTCATCATCTGTTCTCGACAATGCCGCATTATAATGCGATGGAAGCT>999
CsFAD2-3 900>AGACTATGGAATCTTGAACAAGGTGTTCCACAACATCACGGACACACATGTGGCTCATCATCTGTTCTCGACAATGCCACATTATAATGCGATGGAAGCT>999

              *         *         *         *         *         *         *         *         *         *
CsFAD2-1 1000>ACAAAGGCGATAAAGCCAATACTCGGTGACTATTACCAGTTCGACGGAACACCATGGTATGTGGCCATGTATAGGGAGGCAAAGGAGTGTATCTATGTAG>1099
CsFAD2-2 1000>ACAAAGGCGATAAAGCCAATACTCGGTGACTATTACCAGTTCGACGGAACACCATGGTATGTGGCCATGTATAGGGAGGCAAAGGAGTGTATCTATGTAG>1099
CsFAD2-3 1000>ACAAAGGCGATAAAGCCAATACTCGGTGACTACTACCAGTTCGACGGAACACCGTGGTATGTGGCGATGTATAGGGAGGCAAAGGAGTGTATCTATGTAG>1099

              *         *         *         *         *         *
CsFAD2-1 1100>AACCGGACAGGGAAGGTGACAAGAAAGGTGTGTACTGGTACAACAATAAGTTATGA>1155
CsFAD2-2 1100>AACCGGACAGGGAAGGTGACAAGAAAGGTGTGTACTGGTACAACAATAAGTTATGA>1155
CsFAD2-3 1100>AACCGGACAGAGAAGGTGACAAGAAAGGTGTGTACTGGTACAACAATAAGTTATGA>1155
                             GTGACAAGAAAGGTGTGTACTG
                                  ←CsFAD2_Enz_Rev
```

FIGURE S2

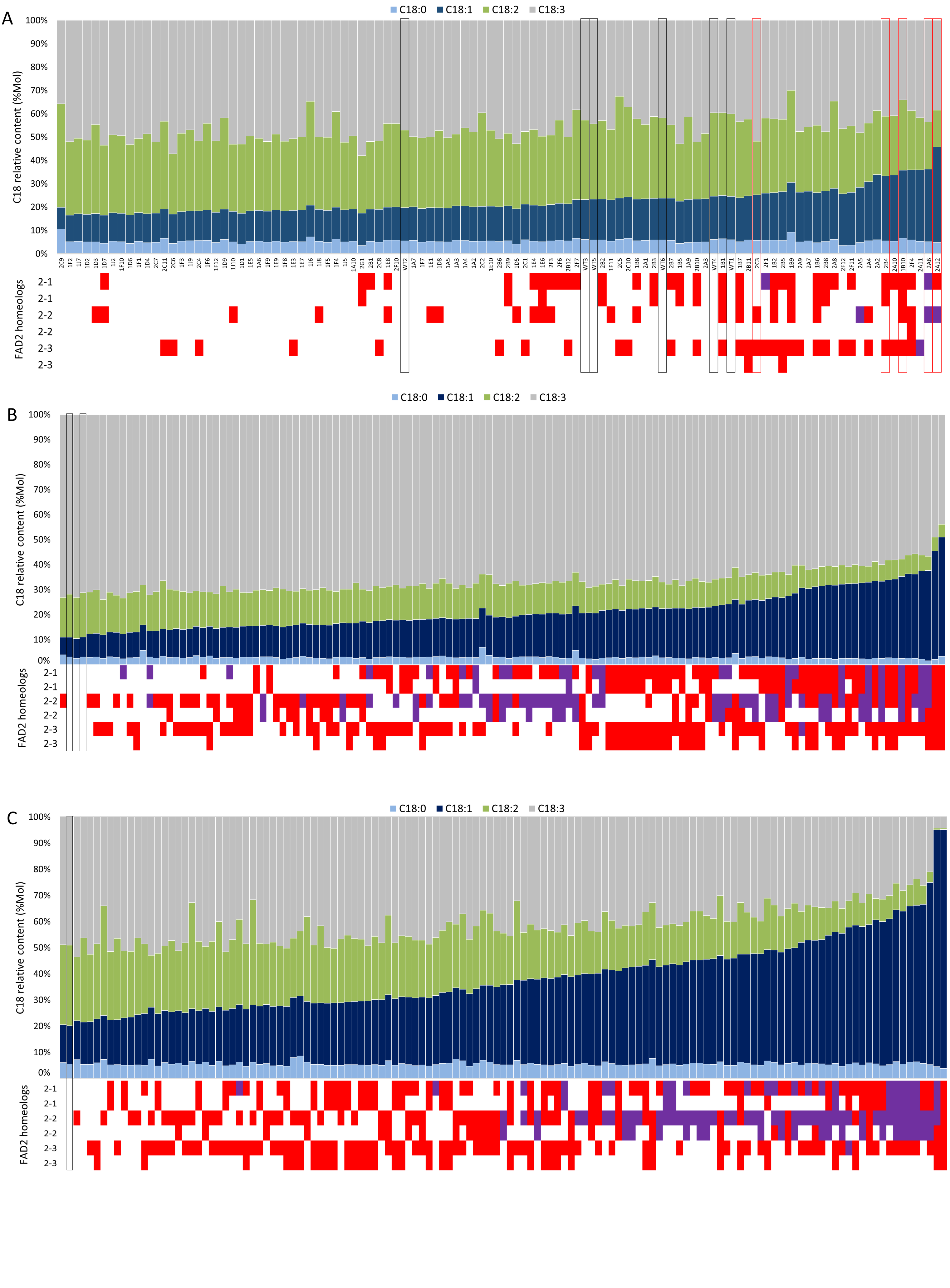
A
B
C
C18:0
C18:1
C18:2
C18:3
C18 relative content (%Mol)
0%
10%
20%
30%
40%
50%
60%
70%
80%
90%
100%
FAD2 homeologs
2-1
2-1
2-2
2-2
2-3
2-3

FIGURE S3

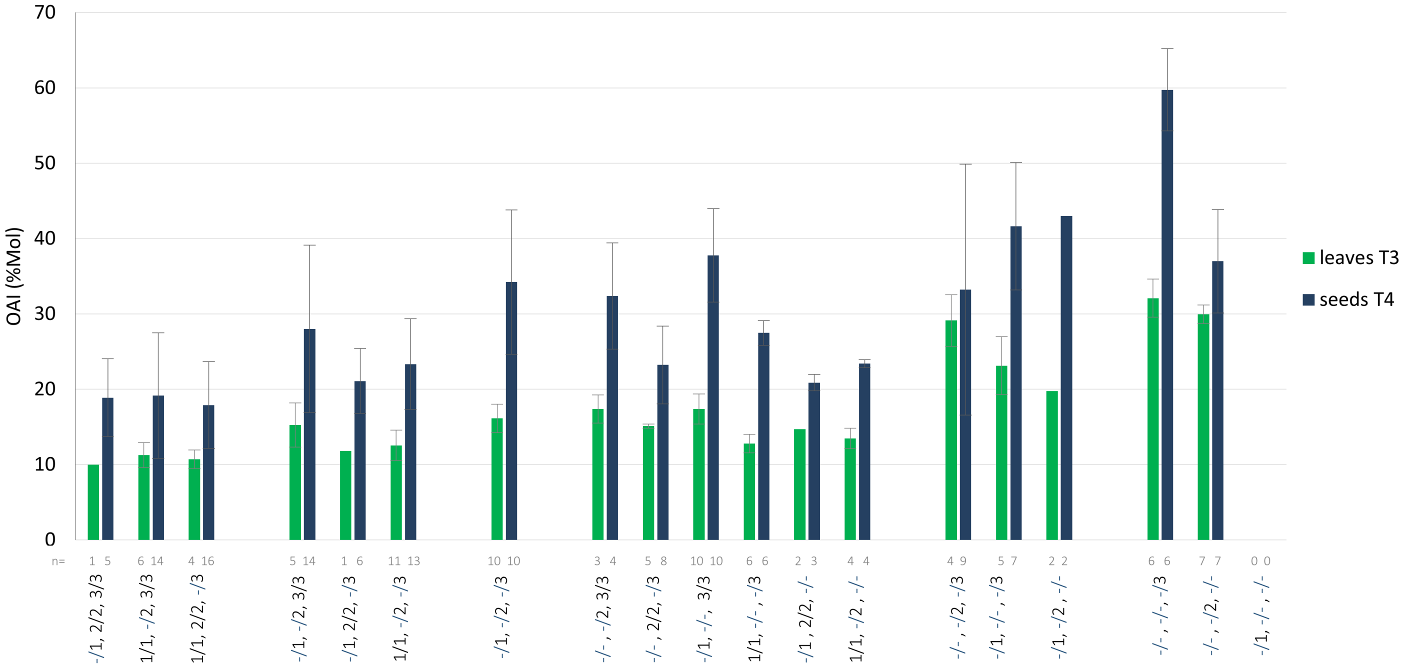
OAI (%Mol)
70
60
50
40
30
20
10
0
leaves T3
seeds T4
n=
1 5
6 14
4 16
5 14
1 6
11 13
10 10
3 4
5 8
10 10
6 6
2 3
4 4
4 9
5 7
2 2
6 6
7 7
0 0
-/1, 2/2, 3/3
1/1, -/2, 3/3
1/1, 2/2, -/3
-/1, -/2, 3/3
-/1, 2/2, -/3
1/1, -/2, -/3
-/1, -/2, -/3
-/- , -/2, 3/3
-/- , 2/2, -/3
-/1, -/-, 3/3
1/1, -/- , -/3
-/1 , 2/2, -/-
1/1, -/2 , -/-
-/- , -/2, -/3
-/1, -/- , -/3
-/1, -/2 , -/-
-/- , -/-, -/3
-/- , -/2, -/-
-/1, -/- , -/-

FIGURE S4

Tissue	CsFAD2-1		CsFAD2-2		CsFAD2-3	
Germinating Seed	15.49	2.82	45.55	1.38	18.56	4.03
Cotyledon	27.19	0.82	53.06	1.26	42.55	2.84
Young Leaf	17.74	0.48	26.64	1.55	26.42	0.4
Senescing Leaf	9.69	4.2	10.24	3.55	7.51	2.23
Root	53.77	6.15	98.78	5.69	58.04	6.02
Stem	37.52	1.67	71.95	2.86	54.82	5.0
Buds	42.96	6.21	91.78	12.45	97.28	13.67
Flower	38.06	1.14	71.55	7.72	67.56	7.25
Early Seed Development	71.97	9.16	242.5	14.42	161.71	21.32
Early-mid Seed Development	182.31	12.71	664.21	84.22	428.7	35.29
Late-mid Seed Development	74.61	17.46	166.24	37.06	139.23	34.41
Late Seed Development	24.89	0.71	34.41	2.02	19.38	1.98

FIGURE S5

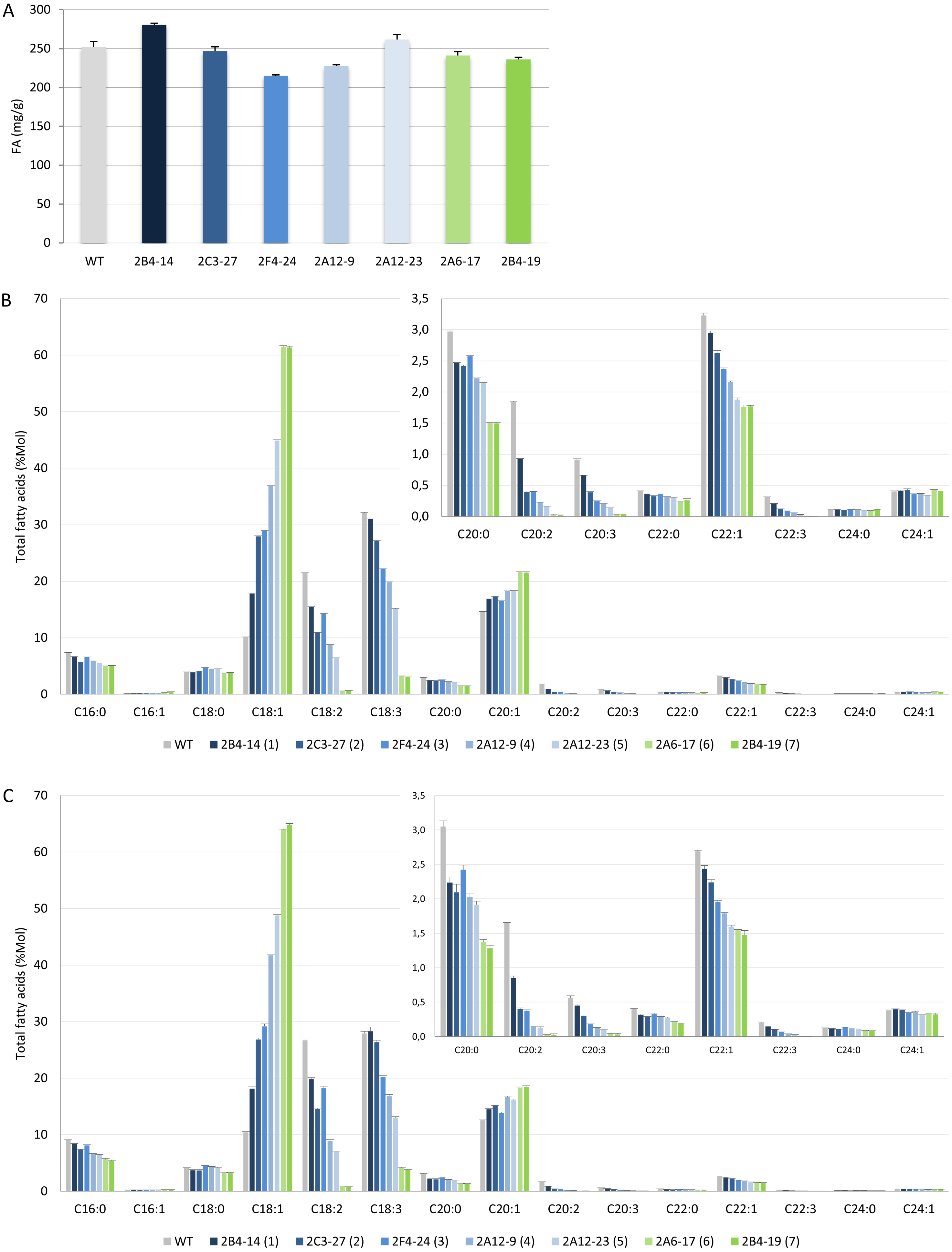
A
FA (mg/g)
0
50
100
150
200
250
300
WT
2B4-14
2C3-27
2F4-24
2A12-9
2A12-23
2A6-17
2B4-19
B
Total fatty acids (%Mol)
0
10
20
30
40
50
60
70
C16:0
C16:1
C18:0
C18:1
C18:2
C18:3
C20:0
C20:1
C20:2
C20:3
C22:0
C22:1
C22:3
C24:0
C24:1
0,0
0,5
1,0
1,5
2,0
2,5
3,0
3,5
WT
2B4-14 (1)
2C3-27 (2)
2F4-24 (3)
2A12-9 (4)
2A12-23 (5)
2A6-17 (6)
2B4-19 (7)
C
Total fatty acids (%Mol)

FIGURE S6

CsU3 promoter:
TTCATTCAACAAGAAAAGAATATGTGTAACTGACGAAAGTTCTTAGGCTA
ACAAAAATGTATTATAGTGAACATAGAAATAACAGAATGCTTTTTTACTC
GTAAACACATGATCACAAAATTCACAATCAAAGTCGGTTTAGATGTACCG
TCCGGTTTAAAGCAAATAAGTTGGGAATGGGTTTTACAAATAAACAAGTG
GGCTTTGGCCCGTATCACCTACTATTACAAGGGAACTCAACTCACATTAC
AATTTACAAGAGTACAAACATCCCACATCGCTCGCCTAGGAAACATAGCT
GTTGTATATATAACGTTGAGAGAGCAACGTTGGTC

CsU6 promoter:
GTTGATGTTTAAGTTATAATTCCCCCATAGGTTCTCGCTAAATCAGATAC
CAGTAACATTTAGCAACATCCCATAGTCTTTTTTTTTTTTAATCAAACTT
TAAACCACAAGTCATTAGCTTTAACAAATTTAAAGCTAACGTTACAAAAC
CAACGGCGTCGTTTCACTCAATCTCTAAGAATAATAATCAGAAAGAGGAC
TTTTAATTACCTTAAATAGGCCTTTTTATCAAACGAGCCCTTTTAAGCTT
TAACCGATAACAAATATACCACATCGCATAGAACAGAGGACAAAAGCTGC
GTTTAAATAATGTTAGAGTCGAGTAAGTGATT

FIGURE S7

Primers		Genes	sequence
CDSFAD2_For145		*FAD2-1, FAD2-2, FAD2-3*	ATCCCTCGCTCTTTCTCCTAC
CsFAD2-FN-Rev		*FAD2-1, FAD2-2, FAD2-3*	TGTAGATGGGAGCGTTGGG
CsFad2a_SNP_R2	SAP primer	*FAD2-1*	TCAAGACGAGGAACGCGT**C(T)T**
CsFad2b_SNP_R	SAP primer	*FAD2-2*	AACCCGTCGTATGGTCTGC**G(C)G**
CsFad2c_SNP_R	SAP primer	*FAD2-3*	CAAGCGAACCCGTCGT**C(A)C**
CsFad2b_SNP_F	SAP primer	*FAD2-2*	GCTGGACGCATCATGATGT**G(T)G**
CsFad2c_SNP_F	SAP primer	*FAD2-3*	TTGTCCCAAAGCAGAAAT**G(C)A**
CsFAD2_Enz_For		*FAD2-1, FAD2-2, FAD2-3*	AACCGATGCCATAAAGCGTG
CsFAD2_Enz_Rev		*FAD2-1, FAD2-2, FAD2-3*	CAGTACACACCTTTCTTGTCAC
CsFAD2_396_For		*FAD2-1, FAD2-2, FAD2-3*	CGTCCCTTACTTCTCCTGG
CDSFAD2_Rev812		*FAD2-1, FAD2-2, FAD2-3*	GGATGAGTGTGCTGCAAGTAAG
CsFAD2_553_Rev		*FAD2-1, FAD2-2, FAD2-3*	ACCCGAGGACAAACTGGAC

TABLE S1
