# Supplementary material for: Selective gene dosage by CRISPR‐Cas9 genome editing in hexaploid Camelina sativa
Source: Plant Biotechnol J. 2017 Apr 1;15(6):729–39. doi: 10.1111/pbi.12671 (PMC5425392; doi:10.1111/pbi.12671)
Supplement: Supplementary file 2 — Table S2. Summary of the different genotypes of CsFAD2 CRISPR T2 and T3 lines. [file PBI-15-729-s002.pdf]

Plant	guide RNA	Generation	Dsred	Gene A				Gene B				Gene C				Plant genotype	OAI	OAI
				A1	A2	Genotype		A1	A2	Genotype		A1	A2	Genotype			T3 or T4 seeds	T3 leaves
1A2	ARNG1	T2	Heterozygote	WT	WT	WT		WT	WT	WT		WT	WT	WT		1/1,2/2,3/3	14,78890099	/
1A3	ARNG1	T2	Homozygote	WT	WT	WT		WT	WT	WT		WT	WT	WT		1/1,2/2,3/3	14,66873316	/
1A4	ARNG1	T2	Heterozygote	WT	WT	WT		WT	WT	WT		WT	WT	WT		1/1,2/2,3/3	14,78000748	/
1A5	ARNG1	T2	Heterozygote	WT	WT	WT		WT	WT	WT		WT	WT	WT		1/1,2/2,3/3	14,58506048	/
1A6	ARNG1	T2	Heterozygote	WT	WT	WT		WT	WT	WT		WT	WT	WT		1/1,2/2,3/3	13,16959454	/
1A7	ARNG1	T2	Homozygote	WT	WT	WT		WT	WT	WT		WT	WT	WT		1/1,2/2,3/3	14,37626115	/
1A9	ARNG1	T2	negative	WT	WT	WT		WT	WT	WT		WT	WT	WT		1/1,2/2,3/3	18,44221558	/
1A10	ARNG1	T2	Heterozygote	WT	WT	WT		WT	WT	WT		WT	WT	WT		1/1,2/2,3/3	13,82616865	/
1B1	ARNG1	T2	Heterozygote	(+A), position 17	(+A), position 17	Homozygote		(+T), position 17	WT	Heterozygote		(+T), position 17	WT	Heterozygote		-/-,-/2,-/3	18,57790781	/
1B2	ARNG1	T2	Heterozygote	(+T), position 17	(+T), position 17	Homozygote		(+T), position 17	WT	Heterozygote		(+T), position 17	WT	Heterozygote		-/-,-/2,-/3	20,3149299	/
1B5	ARNG1	T2	Homozygote	(+T), position 17	(+T), position 17	Homozygote		WT	WT	WT		WT	WT	WT		-/-,2/2,3/3	18,12657104	/
1B6	ARNG1	T2	Heterozygote	(+T), position 17	(+T), position 17	Homozygote		(+T), position 17	WT	Heterozygote		(+T), position 17	WT	Heterozygote		-/-,-/2,-/3	21,47177977	/
1B7	ARNG1	T2	Homozygote	(+T), position 17	(+T), position 17	Homozygote		(+T), position 17	WT	Heterozygote		(+T), position 17	WT	Heterozygote		-/-,-/2,-/3	18,89163727	/
1B8	ARNG1	T2	Heterozygote	(+T), position 17	WT	Heterozygote		(+T), position 17	WT	Heterozygote		WT	WT	WT		-/1,-/2,3/3	17,7471432	/
1B9	ARNG1	T2	Heterozygote	(+T), position 17	(+T), position 17	Homozygote		(+T), position 17	WT	Heterozygote		(+T), position 17	WT	Heterozygote		-/-,-/2,-/3	21,28628772	/
1B10	ARNG1	T2	Homozygote	(+T), position 17	(+T), position 17	Homozygote		(+T), position 17	WT	Heterozygote		WT	WT	WT		-/-,-/2,3/3	29,08801791	/
1D1	ARNG1	T2	Heterozygote	WT	WT	WT		WT	WT	WT		WT	WT	WT		1/1,2/2,3/3	13,14108859	/
1D2	ARNG1	T2	Heterozygote	WT	WT	WT		WT	WT	WT		WT	WT	WT		1/1,2/2,3/3	11,80770545	/
1D3	ARNG1	T2	Heterozygote	WT	WT	WT		(+A), position 17	WT	Heterozygote		WT	WT	WT		1/1,-/2,3/3	12,08323097	/
1D4	ARNG1	T2	Heterozygote	WT	WT	WT		WT	WT	WT		WT	WT	WT		1/1,2/2,3/3	12,39339509	/
1D5	ARNG1	T2	Heterozygote	WT	WT	WT		(+T), position 17	WT	Heterozygote		WT	WT	WT		1/1,-/2,3/3	15,1735965	/
1D6	ARNG1	T2	Heterozygote	WT	WT	WT		WT	WT	WT		WT	WT	WT		1/1,2/2,3/3	12,25465847	/
1D7	ARNG1	T2	Heterozygote	(+T), position 17	WT	Heterozygote		(+T), position 17	WT	Heterozygote		WT	WT	WT		-/1,-/2,3/3	12,09186252	/
1D8	ARNG1	T2	Heterozygote	WT	WT	WT		(+T), position 17	WT	Heterozygote		WT	WT	WT		1/1,-/2,3/3	14,56563491	/
1D9	ARNG1	T2	Heterozygote	WT	WT	WT		WT	WT	WT		WT	WT	WT		1/1,2/2,3/3	13,03769727	/
1D10	ARNG1	T2	Heterozygote	WT	WT	WT		(+A), position 17	WT	Heterozygote		WT	WT	WT		1/1,-/2,3/3	14,40266768	/
1E3	ARNG1	T2	Homozygote	WT	WT	WT		WT	WT	WT		(+A), position 17	WT	Heterozygote		1/1,2/2,-/3	13,3901112	/
1E4	ARNG1	T2	Homozygote	(+T), position 17	WT	Heterozygote		(+T), position 17	WT	Heterozygote		WT	WT	WT		-/1,-/2,3/3	15,41260788	/
1E5	ARNG1	T2	Homozygote	WT	WT	WT		WT	WT	WT		WT	WT	WT		1/1,2/2,3/3	13,16876311	/
1E6	ARNG1	T2	Heterozygote	(+A), position 15	WT	Heterozygote		(+T), position 17	WT	Heterozygote		WT	WT	WT		-/1,-/2,3/3	15,44051321	/
1E7	ARNG1	T2	Homozygote	WT	WT	WT		WT	WT	WT		WT	WT	WT		1/1,2/2,3/3	13,60899435	/
1E8	ARNG1	T2	Heterozygote	(+T), position 17	WT	Heterozygote		(+T), position 17	WT	Heterozygote		WT	WT	WT		-/1,-/2,3/3	14,12882224	/
1E9	ARNG1	T2	Heterozygote	WT	WT	WT		WT	WT	WT		WT	WT	WT		1/1,2/2,3/3	13,3523873	/
1E10	ARNG1	T2	Homozygote	WT	WT	WT		WT	WT	WT		WT	WT	WT		1/1,2/2,3/3	14,9016276	/
1F1	ARNG1	T2	Homozygote	WT	WT	WT		WT	WT	WT		WT	WT	WT		1/1,2/2,3/3	12,30881453	/
1F2	ARNG1	T2	Homozygote	WT	WT	WT		WT	WT	WT		WT	WT	WT		1/1,2/2,3/3	11,30258281	/
1F3	ARNG1	T2	Homozygote	WT	WT	WT		WT	WT	WT		WT	WT	WT		1/1,2/2,3/3	12,62704838	/
1F4	ARNG1	T2	Homozygote	WT	WT	WT		WT	WT	WT		WT	WT	WT		1/1,2/2,3/3	13,70924568	/
1F5	ARNG1	T2	Homozygote	WT	WT	WT		WT	WT	WT		WT	WT	WT		1/1,2/2,3/3	13,70570372	/
1F6	ARNG1	T2	Homozygote	WT	WT	WT		WT	WT	WT		WT	WT	WT		1/1,2/2,3/3	12,96965163	/
1F7	ARNG1	T2	Homozygote	WT	WT	WT		WT	WT	WT		WT	WT	WT		1/1,2/2,3/3	14,39216752	/
1F8	ARNG1	T2	Heterozygote	WT	WT	WT		WT	WT	WT		WT	WT	WT		1/1,2/2,3/3	13,3810763	/
1F9	ARNG1	T2	Homozygote	WT	WT	WT		WT	WT	WT		WT	WT	WT		1/1,2/2,3/3	13,32151111	/
1F10	ARNG1	T2	Homozygote	WT	WT	WT		WT	WT	WT		WT	WT	WT		1/1,2/2,3/3	12,20914606	/
1F11	ARNG1	T2	Heterozygote	WT	WT	WT		(-CC), position 14	WT	Heterozygote		WT	WT	WT		1/1,-/2,3/3	17,70234243	/
1F12	ARNG1	T2	Homozygote	WT	WT	WT		WT	WT	WT		WT	WT	WT		1/1,2/2,3/3	12,98912264	/
1J2	ARNG1	T2	Homozygote	WT	WT	WT		WT	WT	WT		WT	WT	WT		1/1,2/2,3/3	12,13406772	/
1J5	ARNG1	T2	Homozygote	WT	WT	WT		WT	WT	WT		WT	WT	WT		1/1,2/2,3/3	13,78014788	/
1J6	ARNG1	T2	Homozygote	WT	WT	WT		WT	WT	WT		WT	WT	WT		1/1,2/2,3/3	13,62041733	/
1J7	ARNG1	T2	Homozygote	WT	WT	WT		WT	WT	WT		WT	WT	WT		1/1,2/2,3/3	11,80642297	/
1J8	ARNG1	T2	Homozygote	WT	WT	WT		(+T), position 17	WT	Heterozygote		WT	WT	WT		1/1,-/2,3/3	13,6979524	/
1J9	ARNG1	T2	Heterozygote	WT	WT	WT		WT	WT	WT		WT	WT	WT		1/1,2/2,3/3	12,71070669	/
1J10	ARNG1	T2	Homozygote	WT	WT	WT		(+A), position 17	WT	Heterozygote		WT	WT	WT		1/1,-/2,3/3	13,09492626	/
2A1	ARNG2	T2	Heterozygote	WT	WT	WT		WT	WT	WT		WT	WT	WT		1/1,2/2,3/3	17,81191445	/
2A2	ARNG2	T2	Homozygote	WT	WT	WT		WT	WT	WT		WT	WT	WT		1/1,2/2,3/3	27,83951932	/
2A3	ARNG2	T2	Heterozygote	WT	WT	WT		WT	WT	WT		WT	WT	WT		1/1,2/2,3/3	18,51186915	/
2A4	ARNG2	T2	Heterozygote	WT	WT	WT		(-TG), position 15	WT	Heterozygote		(-G), position 14	WT	Heterozygote		1/1,-/2,-/3	25,30515041	/
2A5	ARNG2	T2	Homozygote	WT	WT	WT		(-CGG), position 11	WT	Heterozygote		WT	WT	WT		1/1,-/2,3/3	23,56641822	/
2A6	ARNG2	T2	Homozygote	(-TGG), position 15	WT	Heterozygote		(-GTGGCC), position 14	WT	Heterozygote		WT	WT	WT		-/1,-/2,3/3	31,13268882	/
2A7	ARNG2	T2	Homozygote	WT	WT	WT		WT	WT	WT		WT	WT	WT		1/1,2/2,3/3	21,37070352	/
2A8	ARNG2	T2	Homozygote	(-TCCTCGGGTG), position 7	WT	Heterozygote		WT	WT	WT		WT	WT	WT		-/1,2/2,3/3	21,79854459	/
2A9	ARNG2	T2	Homozygote	WT	WT	WT		WT	WT	WT		(-G), position 14	WT	Heterozygote		1/1,2/2,-/3	21,30948557	/
2A10	ARNG2	T2	Homozygote	(-GG), position 13	WT	Heterozygote		WT	WT	WT		(-GGGTGGCCC), position 12	WT	Heterozygote		-/1,2/2,-/3	28,31970599	/
2A11	ARNG2	T2	Homozygote	WT	WT	WT		WT	WT	WT		(-TGG), position 15	WT	Heterozygote		1/1,2/2,-/3	30,63116405	/
2A12	ARNG2	T2	Homozygote	(+A), position 14	WT	Heterozygote		(-33)	WT	Heterozygote		WT	WT	WT		-/1,-/2,3/3	40,98657189	/
2B1	ARNG2	T2	Heterozygote	(+G), position 13	WT	Heterozygote		WT	WT	WT		WT	WT	WT		-/1,2/2,3/3	13,83905816	/
2B2	ARNG2	T2	Heterozygote	(+G), position 13	(+G), position 13	Homozygote		WT	WT	WT		WT	WT	WT		-/-,2/2,3/3	17,48577431	/
2B3	ARNG2	T2	Heterozygote	(+G), position 13	(+G), position 13	Homozygote		WT	WT	WT		WT	WT	WT		-/-,2/2,3/3	17,82866255	/
2B4	ARNG2	T2	Heterozygote	(+G), position 13	(+G), position 13	Homozygote		WT	WT	WT		(-T), position 15	WT	Heterozygote		-/-,2/2,-/3	27,87846786	/
2B5	ARNG2	T2	Heterozygote	(+G), position 13	WT	Heterozygote		WT	WT	WT		(-T), position 15	(-T), position 15	Homozygote		-/1,2/2,-/-	20,99708881	/
2B6	ARNG2	T2	Heterozygote	WT	WT	WT		WT	WT	WT		(-T), position 15	WT	Heterozygote		1/1,2/2,-/3	14,97649754	/
2B7	ARNG2	T2	Heterozygote	(+G), position 13	WT	Heterozygote		WT	WT	WT		(-T), position 15	WT	Heterozygote		-/1,2/2,-/3	18,06804416	/
2B8	ARNG2	T2	Heterozygote	(+G), position 13	(+G), position 13	Homozygote		WT	WT	WT		(-T), position 15	WT	Heterozygote		-/-,2/2,-/3	21,62651324	/
2B9	ARNG2	T2	Heterozygote	(+G), position 13	(+G), position 13	Homozygote		WT	WT	WT		WT	WT	WT		-/-,2/2,3/3	15,03298808	/
2B10	ARNG2	T2	Heterozygote	(+G), position 13	(+G), position 13	Homozygote		WT	WT	WT		(-T), position 15	WT	Heterozygote		-/-,2/2,-/3	18,45356833	/
2B11	ARNG2	T2	Heterozygote	WT	WT	WT		WT	WT	WT		(-T), position 15	(-T), position 15	Homozygote		1/1,2/2,-/-	18,93996173	/
2B12	ARNG2	T2	Heterozygote	(+G), position 13	WT	Heterozygote		WT	WT	WT		(-T), position 15	WT	Heterozygote		-/1,2/2,-/3	15,86240973	/
2C1	ARNG2	T2	negative	WT	WT	WT		WT	WT	WT		(+T), position 14	WT	Heterozygote		1/1,2/2,-/3	15,1968293	/
2C2	ARNG2	T2	negative	WT	WT	WT		WT	WT	WT		WT	WT	WT		1/1,2/2,3/3	14,83160685	/
2C3	ARNG2	T2	negative	WT	WT	WT		(-G), position 14	WT	Heterozygote		(+T), position 14	WT	Heterozygote		1/1,-/2,-/3	19,49721873	/
2C4	ARNG2	T2	negative	WT	WT	WT		WT	WT	WT		(+T), position 14	WT	Heterozygote		1/1,2/2,-/3	12,71340162	/
2C5	ARNG2	T2	negative	WT	WT	WT		WT	WT	WT		(+T), position 14	WT	Heterozygote		1/1,2/2,-/3	17,71664078	/
2C6	ARNG2	T2	negative	WT	WT	WT		WT	WT	WT		(+T), position 14	WT	Heterozygote		1/1,2/2,-/3	12,58542171	/
2C7	ARNG2	T2	negative	WT	WT	WT		WT	WT	WT		WT	WT	WT		1/1,2/2,3/3	12,51060455	/
2C8	ARNG2	T2	negative	WT	WT	WT		WT	WT	WT		(+T), position 14	WT	Heterozygote		1/1,2/2,-/3	14,02684587	/
2C9	ARNG2	T2	negative	WT	WT	WT		WT	WT	WT		WT	WT	WT		1/1,2/2,3/3	9,277850839	/

2C10	ARNG2	T2	Heterozygote	WT	WT	WT		WT	WT	WT		(+T), position 14	WT	Heterozygote		1/1,2/2,-/3	17,71828142	/
2C11	ARNG2	T2	Heterozygote	WT	WT	WT		WT	WT	WT		(+T), position 14	WT	Heterozygote		1/1,2/2,-/3	12,55183416	/
2F1	ARNG2	T2	Heterozygote	(-TGG), position 15	WT	Heterozygote		WT	WT	WT		(-CGGGTGGCCCT), position 11	WT	Heterozygote		-/1,2/2,-/3	20,15256802	/
2F3	ARNG2	T2	Heterozygote	(+G), position 13	WT	Heterozygote		(-G), position 14	WT	Heterozygote		WT	WT	WT		-/1,-/2,3/3	15,5522449	/
2F4	ARNG2	T2	Heterozygote	(-G), position 14	WT	Heterozygote		(-TCCTCGGGTG), position 7	(-G), position 14	Homozygote		(-G), position 14	WT	Heterozygote		-/1,-/-,-/3	30,03413417	/
2F6	ARNG2	T2	Heterozygote	(+G), position 13	WT	Heterozygote		WT	WT	WT		WT	WT	WT		-/1,2/2,3/3	15,69099619	/
2F7	ARNG2	T2	Heterozygote	(+G), position 13	WT	Heterozygote		WT	WT	WT		WT	WT	WT		-/1,2/2,3/3	16,60332782	/
2F10	ARNG2	T2	Homozygote	WT	WT	WT		WT	WT	WT		WT	WT	WT		1/1,2/2,3/3	14,20645417	/
2F11	ARNG2	T2	Heterozygote	(+G), position 13	WT	Heterozygote		WT	WT	WT		(-G), position 14	WT	Heterozygote		-/1,2/2,-/3	22,63951033	/
2F12	ARNG2	T2	Heterozygote	WT	WT	WT		WT	WT	WT		(-G), position 14	WT	Heterozygote		1/1,2/2,-/3	22,18082935	/
2G1	ARNG2	T2	Heterozygote	(+G), position 13	(+G), position 13	Homozygote		WT	WT	WT		WT	WT	WT		-/-,2/2,3/3	13,83601661	/
2A6.1	ARNG2	T3	Homozygote	(-TGG), position 15	(-TGG), position 15	Homozygote		(-GTGGCC), position 14	(-GTGGCC), position 14	Homozygote		WT	(-40), position -23 a +17	Heterozygote		-/-,-/-,-/3	59,66878155	30,63990946
2A6.3	ARNG2	T3	Homozygote	(-TGG), position 15	WT	Heterozygote		(-GTGGCC), position 14	WT	Heterozygote		WT	WT	WT		-/1,-/2,3/3	33,70861837	10,37366592
2A6.4	ARNG2	T3	Homozygote	(-TGG), position 15	(-TGG), position 15	Homozygote		(-GTGGCC), position 14	WT	Heterozygote		WT	WT	WT		-/-,-/2,3/3	38,33109431	19,04962062
2A6.5	ARNG2	T3	Homozygote	(-TGG), position 15	WT	Heterozygote		(-GTGGCC), position 14	(-GTGGCC), position 14	Homozygote		(-G), position 14	WT	Heterozygote		-/1,-/-,-/3	47,4923141	21,29150763
2A6.6	ARNG2	T3	Homozygote	(-TGG), position 15	(-TGG), position 15	Homozygote		(-GTGGCC), position 14	(-GTGGCC), position 14	Homozygote		(-40), position -23 a +17	WT	Heterozygote		-/-,-/-,-/3	59,8065588	30,97171255
2A6.7	ARNG2	T3	Homozygote	(-TGG), position 15	WT	Heterozygote		(-GTGGCC), position 14	WT	Heterozygote		(-40), position -23 a +17	WT	Heterozygote		-/1,-/2,-/3	41,72936991	15,14968525
2A6.8	ARNG2	T3	Homozygote	(-TGG), position 15	WT	Heterozygote		(-GTGGCC), position 14	WT	Heterozygote		(-40), position -23 a +17	WT	Heterozygote		-/1,-/2,-/3	32,197141	15,50651448
2A6.9	ARNG2	T3	Homozygote	(-TGG), position 15	(-TGG), position 15	Homozygote		(-GTGGCC), position 14	WT	Heterozygote		(-17), position 8	(+G), position 15	Homozygote		-/-,-/2,-/-	50,2607327	29,65727067
2A6.10	ARNG2	T3	Homozygote	(-TGG), position 15	WT	Heterozygote		(-GTGGCC), position 14	(-GTGGCC), position 14	Homozygote		WT	WT	WT		-/1,-/-,3/3	43,86717908	17,63576444
2A6.11	ARNG2	T3	Homozygote	(-TGG), position 15	(-TGG), position 15	Homozygote		WT	WT	WT		(-40), position -23 a +17	WT	Heterozygote		-/-,2/2,-/3	33,98019777	15,52194956
2A6.13	ARNG2	T3	Homozygote	(-TGG), position 15	(-TGG), position 15	Homozygote		(-GTGGCC), position 14	WT	Heterozygote		WT	WT	WT		-/-,-/2,3/3	38,07239058	17,67244118
2A6.14	ARNG2	T3	Homozygote	(-TGG), position 15	(+G), position 15	Homozygote		WT	WT	WT		(-40), position -23 a +17	(-40), position -23 a +17	Homozygote		-/-,2/2,-/-	42,65074683	26,11640659
2A6.15	ARNG2	T3	Homozygote	WT	WT	WT		(-GTGGCC), position 14	WT	Heterozygote		(-40), position -23 a +17	WT	Heterozygote		1/1,-/2,-/3	34,22514965	14,87743533
2A6.16	ARNG2	T3	Homozygote	(-TGG), position 15	WT	Heterozygote		(-GTGGCC), position 14	WT	Heterozygote		(-40), position -23 a +17	WT	Heterozygote		-/1,-/2,-/3	35,86478041	14,60932182
2A6.17	ARNG2	T3	Homozygote	(-TGG), position 15	(-TGG), position 15	Homozygote		(-GTGGCC), position 14	(+G), position 15	Homozygote		(-40), position -23 a +17	(-40), position -23 a +17	Homozygote		-/-,-/-,-/-	90,65548861	36,14342974
2A6.18	ARNG2	T3	Homozygote	(-TGG), position 15	WT	Heterozygote		WT	WT	WT		WT	WT	WT		-/1,2/2,3/3	26,46518145	9,96827493
2A6.19	ARNG2	T3	Homozygote	(-TGG), position 15	WT	Heterozygote		(-GTGGCC), position 14	WT	Heterozygote		(-40), position -23 a +17	WT	Heterozygote		-/1,-/2,-/3	47,00443516	18,58139294
2A6.20	ARNG2	T3	Homozygote	(-CTCGG), position 9	WT	Heterozygote		(-GTGGCC), position 14	(-GT), position 14	Homozygote		(-40), position -23 a +17	WT	Heterozygote		-/1,-/-,-/3	47,09131538	21,69339262
2A6.21	ARNG2	T3	Homozygote	(-TGG), position 15	(-TGG), position 15	Homozygote		(-GTGGCC), position 14	(-GTGGCC), position 14	Homozygote		(-40), position -23 a +17	WT	Heterozygote		-/-,-/-,-/3	58,08672652	35,21498659
2A6.22	ARNG2	T3	Homozygote	(-TGG), position 15	(-TGG), position 15	Homozygote		(-GTGGCC), position 14	(-GTGGCC), position 14	Homozygote		(-40), position -23 a +17	WT	Heterozygote		-/-,-/-,-/3	58,40901852	32,759969
2A6.23	ARNG2	T3	Homozygote	(-TGG), position 15	(-TGG), position 15	Homozygote		(-GTGGCC), position 14	WT	Heterozygote		(-40), position -23 a +17	WT	Heterozygote		-/-,-/2,-/3	55,77421695	25,1336359
2A6.24	ARNG2	T3	Homozygote	(-TGG), position 15	WT	Heterozygote		(-GTGGCC), position 14	WT	Heterozygote		WT	WT	WT		-/1,-/2,3/3	35,41300011	16,2381076
2A6.25	ARNG2	T3	Homozygote	(-TGG), position 15	WT	Heterozygote		(-GTGGCC), position 14	(-GTGGCC), position 14	Homozygote		WT	WT	WT		-/1,-/-,3/3	39,1695296	16,10653097
2A6.26	ARNG2	T3	Homozygote	(-TGG), position 15	WT	Heterozygote		(-GTGGCC), position 14	(-G), position 14	Homozygote		WT	WT	WT		-/1,-/-,3/3	42,99765213	21,48454488
2A12.1	ARNG2	T3	Homozygote	(+A), position 14	WT	Heterozygote		(-33), position 11	WT	Heterozygote		WT	WT	WT		-/1,-/2,3/3	37,60163596	NA
2A12.2	ARNG2	T3	Homozygote	WT	WT	WT		(-33), position 11	WT	Heterozygote		WT	WT	WT		1/1,-/2,3/3	34,31155594	13,20413265
2A12.4	ARNG2	T3	Homozygote	WT	WT	WT		(-33), position 11	(-33), position 11	Homozygote		WT	WT	WT		1/1,-/-,3/3	37,26097831	12,59700717
2A12.5	ARNG2	T3	Homozygote	(+A), position 14	WT	Heterozygote		(-18), position 6	(-18), position 6	Homozygote		WT	WT	WT		-/1,-/-,3/3	37,50303247	14,97166947
2A12.6	ARNG2	T3	Homozygote	(+A), position 14	(-G), position 14	Homozygote		(-33), position 11	WT	Heterozygote		(-TGG), position 15	WT	Heterozygote		-/-,-/2,-/3	52,2857853	27,68122395
2A12.8	ARNG2	T3	Homozygote	(+A), position 14	WT	Heterozygote		(-33), position 11	(-33), position 11	Homozygote		WT	WT	WT		-/1,-/-,3/3	37,89403458	17,85326031
2A12.9	ARNG2	T3	Homozygote	(+A), position 14	(+A), position 14	Homozygote		(-18), position 6	(-18), position 6	Homozygote		WT	WT	WT		-/-,-/-,3/3	52,89171317	28,94843198
2A12.10	ARNG2	T3	Homozygote	(+A), position 14	WT	Heterozygote		(-33), position 11	(-33), position 11	Homozygote		WT	WT	WT		-/1,-/-,3/3	40,09822537	17,61381112
2A12.11	ARNG2	T3	Homozygote	(+A), position 14	WT	Heterozygote		(-33), position 11	WT	Heterozygote		WT	WT	WT		-/1,-/2,3/3	41,02088321	17,34029631
2A12.12	ARNG2	T3	Homozygote	(+A), position 14	(+A), position 14	Homozygote		(-33), position 11	(-33), position 11	Homozygote		(-TTGTCCTCGG), position 4	WT	Heterozygote		-/-,-/-,-/3	69,65967219	29,29089836
2A12.13	ARNG2	T3	Homozygote	(+A), position 14	WT	Heterozygote		(-33), position 11	(-33), position 11	Homozygote		WT	WT	WT		-/1,-/-,3/3	43,12129098	20,5289542
2A12.14	ARNG2	T3	Homozygote	WT	WT	WT		(-18), position 6	(-18), position 6	Homozygote		WT	WT	WT		1/1,-/-,3/3	32,56444121	16,01292197
2A12.15	ARNG2	T3	Homozygote	(+A), position 14	WT	Heterozygote		(-33), position 11	WT	Heterozygote		(-CCTCGGGT), position 8	WT	Heterozygote		-/1,-/2,-/3	46,891644	22,63161842
2A12.16	ARNG2	T3	Homozygote	(+A), position 14	(+A), position 14	Homozygote		(-33), position 11	WT	Heterozygote		(-TG), position 15	WT	Heterozygote		-/-,-/2,-/3	55,07622722	33,7107784
2A12.17	ARNG2	T3	Homozygote	(+A), position 14	WT	Heterozygote		(-33), position 11	WT	Heterozygote		WT	WT	WT		-/1,-/2,3/3	39,41141039	16,76781188
2A12.18	ARNG2	T3	Homozygote	(+A), position 14	WT	Heterozygote		(-18), position 6	(-18), position 6	Homozygote		WT	WT	WT		-/1,-/-,3/3	39,41141039	16,91364155
2A12.19	ARNG2	T3	Homozygote	(-G), position 14	WT	Heterozygote		(-33), position 11	WT	Heterozygote		WT	WT	WT		-/1,-/2,3/3	42,56528181	15,52040899
2A12.21	ARNG2	T3	Homozygote	(-TG), position 15	WT	Heterozygote		(-18), position 6	(-18), position 6	Homozygote		(-GTGGC), position 14	WT	Heterozygote		-/1,-/-,-/3	42,61360665	22,82664774
2A12.22	ARNG2	T3	Homozygote	(+A), position 14	(+A), position 14	Homozygote		(-18), position 6	(-18), position 6	Homozygote		WT	WT	WT		-/-,-/-,3/3	49,53009073	23,94833506
2A12.23	ARNG2	T3	Homozygote	(+A), position 14	(+A), position 14	Homozygote		(-18), position 6	(-18), position 6	Homozygote		WT	WT	WT		-/-,-/-,3/3	60,79347837	30,37671828
2A12.24	ARNG2	T3	Homozygote	WT	WT	WT		(-33), position 11	WT	Heterozygote		WT	WT	WT		1/1,-/-,3/3	40,40277388	17,05563929
2A12.25	ARNG2	T3	Homozygote	WT	WT	WT		(-33), position 11	WT	Heterozygote		(+G), position 15	WT	Heterozygote		1/1,-/2,-/3	38,42168177	15,7854765
2A12.26	ARNG2	T3	Homozygote	(+A), position 14	(+A), position 14	Homozygote		(-18), position 6	(-18), position 6	Homozygote		WT	WT	WT		-/-,-/-,3/3	55,18443486	23,21763386
2A12.27	ARNG2	T3	Homozygote	(-GG), position 13	WT	Heterozygote		(-18), position 6	(-18), position 6	Homozygote		(-GTGGC), position 14	WT	Heterozygote		-/1,-/-,-/3	50,74721902	NA
2A12.29	ARNG2	T3	Homozygote	WT	WT	WT		(-33), position 11	(-33), position 11	Homozygote		WT	WT	WT		1/1,-/-,3/3	30,59062825	13,65583361
2A12.30	ARNG2	T3	Homozygote	(-G), position 14	WT	Heterozygote		(-33), position 11	WT	Heterozygote		(-G), position 14	WT	Heterozygote		-/1,-/2,-/3	40,65400633	17,43768303
2B4.1	ARNG2	T3	negative	(+G), position 15	(+G), position 15	Homozygote		WT	WT	WT		(-T), position 15	(-T), position 15	Homozygote		-/-,2/2,-/-	25,62080228	19,19420017
2B4.2	ARNG2	T3	negative	(+G), position 15	(+G), position 15	Homozygote		WT	WT	WT		(-T), position 15	(-T), position 15	Homozygote		-/-,2/2,-/-	26,02185534	19,2287373
2B4.3	ARNG2	T3	negative	(+G), position 15	(+G), position 15	Homozygote		(-TG), position 15	WT	Heterozygote		(-GTGG,+78), position 14	(-TGG,+77), position 15	Homozygote		-/-,-/2,-/-	41,22019322	31,53752127
2B4.4	ARNG2	T3	negative	(+G), position 15	(+G), position 15	Homozygote		(-TG), position 15	WT	Heterozygote		(-GTGG,+78), position 14	(-TGG,+77), position 15	Homozygote		-/-,-/2,-/-	33,25679721	30,8020575
2B4.5	ARNG2	T3	negative	(+G), position 15	(+G), position 15	Homozygote		WT	WT	WT		(-GTGG,+78), position 14	(-TGG,+77), position 15	Homozygote		-/-,2/2,-/-	23,92363512	19,4736724
2B4.6	ARNG2	T3	negative	(+G), position 15	(+G), position 15	Homozygote		WT	WT	WT		(-T), position 15	WT	Heterozygote		-/-,2/2,-/3	22,25415272	14,9051144
2B4.7	ARNG2	T3	negative	(+G), position 15	(+G), position 15	Homozygote		(-TG), position 15	WT	Heterozygote		(-T), position 15	WT	Heterozygote		-/-,-/2,-/3	35,3783072	29,9756576
2B4.8	ARNG2	T3	negative	(+G), position 15	(+G), position 15	Homozygote		WT	WT	WT		(-T), position 15	(-T), position 15	Homozygote		-/-,2/2,-/-	27,28372606	19,62930317
2B4.9	ARNG2	T3	negative	(+G), position 15	(+G), position 15	Homozygote		(-TG), position 15	(-TG), position 15	Homozygote		(-GTGG,+78), position 14	(-TGG,+77), position 15	Homozygote		-/-,-/-,-/-		43,26965566
2B4.10	ARNG2	T3	negative	(+G), position 15	(+G), position 15	Homozygote		(-TG), position 15	WT	Heterozygote		(-T), position 15	(-T), position 15	Homozygote		-/-,-/2,-/-	33,51207567	29,62121098
2B4.11	ARNG2	T3	negative	(+G), position 15	(+G), position 15	Homozygote		WT	WT	WT		WT	WT	WT		-/-,2/2,3/3	18,12294044	12,53583542
2B4.12	ARNG2	T3	negative	(+G), position 15	(+G), position 15	Homozygote		WT	WT	WT		(-T), position 15	(-T), position 15	Homozygote		-/-,2/2,-/-	23,7699339	19,34656073
2B4.13	ARNG2	T3	negative	(+G), position 15	(+G), position 15	Homozygote		(-TG), position 15	WT	Heterozygote		(-T), position 15	(-T), position 15	Homozygote		-/-,-/2,-/-	32,26273265	28,74720906
2B4.14	ARNG2	T3	negative	(+G), position 15	(+G), position 15	Homozygote		WT	WT	WT		(-T), position 15	(-T), position 15	Homozygote		-/-,2/2,-/-	24,57513965	19,26572202
2B4.15	ARNG2	T3	negative	(+G), position 15	(+G), position 15	Homozygote		(-TG), position 15	WT	Heterozygote		(-GTGG,+78), position 14	(-TGG,+77), position 15	Homozygote		-/-,-/2,-/-	37,64388838	28,21508944
2B4.16	ARNG2	T3	negative	(+G), position 15	(+G), position 15	Homozygote		WT	WT	WT		(-T), position 15	(-T), position 15	Homozygote		-/-,2/2,-/-	22,86297898	19,40152444
2B4.18	ARNG2	T3	negative	(+G), position 15	(+G), position 15	Homozygote		WT	WT	WT		(-T), position 15	WT	Heterozygote		-/-,2/2,-/3	21,25968564	15,29482049
2B4.19	ARNG2	T3	negative	(+G), position 15	(+G), position 15	Homozygote		(-TG), position 15	(-TG), position 15	Homozygote		(-GTGG,+78), position 14	(-TGG,+77), position 15	Homozygote		-/-,-/-,-/-	91,33197631	47,66636047
2B4.20	ARNG2	T3	negative	(+G), position 15	(+G), position 15	Homozygote		WT	WT	WT		(-T), position 15	WT	Heterozygote		-/-,2/2,-/3	19,97216412	14,87608522
2B4.21	ARNG2	T3	negative	(+G), position 15	(+G), position 15	Homozygote		WT	WT	WT		WT	WT	WT		-/-,2/2,3/3	17,14636407	12,67671007
2B4.22	ARNG2	T3	negative	(+G), position 15	(+G), position 15	Homozygote		WT	WT	WT		(-T), position 15	(-T), position 15	Homozygote		-/-,2/2,-/-	27,0982779	23,01779397
2B4.24	ARNG2	T3	negative	(+G), position 15	(+G), position 15	Homozygote		WT	WT	WT		(-GTGG,+78), position 14	(-TGG,+77), position 15	Homozygote		-/-,2/2,-/-	24,05299043	19,64813201
2B4.26	ARNG2	T3	negative	(+G), position 15	(+G), position 15	Homozygote		WT	WT	WT		(-GTGG,+78), position 14	(-TGG,+77), position 15	Homozygote		-/-,2/2,-/-	25,41599645	19,92261442
2B4.27	ARNG2	T3	negative	(+G), position 15	(+G), position 15	Homozygote		WT	WT	WT		(-GTGG,+78), position 14	(-GTGG,+78), position 14	Homozygote		-/-,2/2,-/-	23,45287009	19,52061468
2B4.28	ARNG2	T3	negative	(+G), position 15	(+G), position 15	Homozygote		(-TG), position 15	WT	Heterozygote		(-GTGG,+78), position 14	(-TGG,+77), position 15	Homozygote		-/-,-/2,-/-	30,74962871	31,12513014
2C3.1	ARNG2	T3	negative	WT	WT	WT		(-G), position 14	(-G), position 14	Homozygote		WT	WT	WT		1/1,-/-,3/3	22,62645661	11,89629597
2C3.2	ARNG2	T3	negative	WT	WT	WT		(-G), position 14	(-G), position 14	Homozygote		(+T), position 15	WT	Heterozygote		1/1,-/-,-/3	27,30731516	12,42291333

2C3.3	ARNG2	T3	negative	WT	WT	WT		(-G), position 14	WT	Heterozygote		(+T), position 15	WT	Heterozygote		1/1,-/2,-/3	19,97566545	14,15733131
2C3.4	ARNG2	T3	negative	WT	WT	WT		(-G), position 14	(-G), position 14	Homozygote		(+T), position 15	(+T), position 15	Homozygote		1/1,-/-,-/-	37,8106971	19,94244795
2C3.5	ARNG2	T3	negative	WT	WT	WT		(-G), position 14	WT	Heterozygote		(+T), position 15	(+T), position 15	Homozygote		1/1,-/2,-/-	22,9688742	11,81212029
2C3.6	ARNG2	T3	negative	WT	WT	WT		WT	WT	WT		WT	WT	WT		1/1,2/2,3/3	16,25267059	7,947667899
2C3.7	ARNG2	T3	negative	WT	WT	WT		(-G), position 14	WT	Heterozygote		(+T), position 15	WT	Heterozygote		1/1,-/2,-/3	21,91513584	9,444407428
2C3.8	ARNG2	T3	negative	WT	WT	WT		(-G), position 14	WT	Heterozygote		(+T), position 15	(+T), position 15	Homozygote		1/1,-/2,-/-	23,00849387	13,67148734
2C3.10	ARNG2	T3	negative	WT	WT	WT		WT	WT	WT		(+T), position 15	WT	Heterozygote		1/1,2/2,-/3	16,87534834	12,06909581
2C3.11	ARNG2	T3	negative	WT	WT	WT		WT	WT	WT		(+T), position 15	WT	Heterozygote		1/1,2/2,-/3	16,37553123	11,44337424
2C3.12	ARNG2	T3	negative	WT	WT	WT		WT	WT	WT		(+T), position 15	WT	Heterozygote		1/1,2/2,-/3	30,82484066	9,459201543
2C3.13	ARNG2	T3	negative	WT	WT	WT		(-G), position 14	WT	Heterozygote		WT	WT	WT		1/1,-/2,3/3	16,85743802	6,992823613
2C3.14	ARNG2	T3	negative	WT	WT	WT		(-G), position 14	WT	Heterozygote		(+T), position 15	WT	Heterozygote		1/1,-/2,-/3	20,16410788	11,46295665
2C3.15	ARNG2	T3	negative	WT	WT	WT		(-G), position 14	WT	Heterozygote		(+T), position 15	WT	Heterozygote		1/1,-/2,-/3	19,97916831	12,83902784
2C3.16	ARNG2	T3	negative	WT	WT	WT		(-G), position 14	(-G), position 14	Homozygote		(+T), position 15	WT	Heterozygote		1/1,-/-,-/3	29,29771166	13,41178943
2C3.17	ARNG2	T3	negative	WT	WT	WT		WT	WT	WT		(+T), position 15	(+T), position 15	Homozygote		1/1,2/2,-/-	16,76365652	10,16560868
2C3.18	ARNG2	T3	negative	WT	WT	WT		(-G), position 14	WT	Heterozygote		(+T), position 15	WT	Heterozygote		1/1,-/2,-/3	22,67278092	10,09576633
2C3.19	ARNG2	T3	negative	WT	WT	WT		(-G), position 14	(-G), position 14	Homozygote		WT	WT	WT		1/1,-/-,3/3	20,47537764	12,72093849
2C3.20	ARNG2	T3	negative	WT	WT	WT		(-G), position 14	WT	Heterozygote		WT	WT	WT		1/1,-/2,3/3	19,1376481	9,392789633
2C3.21	ARNG2	T3	negative	WT	WT	WT		(-G), position 14	WT	Heterozygote		(+T), position 15	WT	Heterozygote		1/1,-/2,-/3	19,81551287	15,34416682
2C3.22	ARNG2	T3	negative	WT	WT	WT		(-G), position 14	(-G), position 14	Homozygote		WT	WT	WT		1/1,-/-,3/3	25,13341056	12,94540652
2C3.23	ARNG2	T3	negative	WT	WT	WT		WT	WT	WT		WT	WT	WT		1/1,2/2,3/3	14,49101151	10,25853968
2C3.24	ARNG2	T3	negative	WT	WT	WT		(-G), position 14	WT	Heterozygote		WT	WT	WT		1/1,-/2,3/3	18,42356889	10,86216504
2C3.25	ARNG2	T3	negative	WT	WT	WT		(-G), position 14	(-G), position 14	Homozygote		WT	WT	WT		1/1,-/-,3/3	23,26407528	11,09108963
2C3.26	ARNG2	T3	negative	WT	WT	WT		(-G), position 14	WT	Heterozygote		(+T), position 15	WT	Heterozygote		1/1,-/2,-/3	20,75304033	10,88868727
2C3.27	ARNG2	T3	negative	WT	WT	WT		(-G), position 14	(-G), position 14	Homozygote		(+T), position 15	(+T), position 15	Homozygote		1/1,-/-,-/-	34,1834576	21,63168415
2C3.28	ARNG2	T3	negative	WT	WT	WT		(-G), position 14	WT	Heterozygote		WT	WT	WT		1/1,-/2,3/3	14,90162014	9,94817544
2C3.29	ARNG2	T3	negative	WT	WT	WT		(-G), position 14	(-G), position 14	Homozygote		(+T), position 15	WT	Heterozygote		1/1,-/-,-/3	28,64772436	12,18379047
2C3.30	ARNG2	T3	negative	WT	WT	WT		(-G), position 14	(-G), position 14	Homozygote		(+T), position 15	WT	Heterozygote		1/1,-/-,-/3	25,18070304	12,39845294
2C3.31	ARNG2	T3	negative	WT	WT	WT		(-G), position 14	(-G), position 14	Homozygote		WT	WT	WT		1/1,-/-,3/3	20,04815404	13,32186571
2F4.1	ARNG2	T3	Heterozygote	WT	WT	WT		WT	WT	WT		(-G), position 14	WT	Heterozygote		1/1,2/2,-/3	17,18976901	9,848916681
2F4.3	ARNG2	T3	Heterozygote	(-G), position 14	(-G), position 14	Homozygote		WT	WT	WT		(-G), position 14	(-G), position 14	Homozygote		-/-,2/2,-/-	24,43917623	20,03049717
2F4.4	ARNG2	T3	Homozygote	(-G), position 14	WT	Heterozygote		WT	WT	WT		(-17), position 8	WT	Heterozygote		-/1,2/2,-/3	21,51610251	11,79967468
2F4.5	ARNG2	T3	Homozygote	(-G), position 14	WT	Heterozygote		WT	WT	WT		(-TG), position 15	(-TG), position 15	Homozygote		-/1,2/2,-/-	19,73227897	14,6165134
2F4.6	ARNG2	T3	Homozygote	(-TGG), position 15	WT	Heterozygote		(-TCCTCGGGTG), position7	WT	Heterozygote		(-G), position 14	WT	Heterozygote		-/1,-/2,-/3	21,90001378	12,10491151
2F4.7	ARNG2	T3	Homozygote	(-G), position 14	(-G), position 14	Homozygote		(-TCCTCGGGTG), position7	WT	Heterozygote		WT	WT	WT		-/-,-/2,3/3	24,00208089	15,3619573
2F4.8	ARNG2	T3	Heterozygote	(-G), position 14	WT	Heterozygote		(-TCCTCGGGTG), position7	(-TCCTCGGGTG), position7	Homozygote		(-G), position 14	WT	Heterozygote		-/1,-/-,-/3	29,67654291	19,89829522
2F4.9	ARNG2	T3	Homozygote	(-G), position 14	WT	Heterozygote		(-TCCTCGGGTG), position7	WT	Heterozygote		(-G), position 14	WT	Heterozygote		-/1,-/2,-/3	25,88539331	16,3383503
2F4.10	ARNG2	T3	Heterozygote	(-G), position 14	(-G), position 14	Homozygote		WT	WT	WT		(-G), position 14	(-G), position 14	Homozygote		-/-,2/2,-/-	24,916418	18,39545869
2F4.11	ARNG2	T3	Homozygote	(-G), position 14	WT	Heterozygote		WT	WT	WT		(-G), position 14	(-TG), position 15	Homozygote		-/1,2/2,-/-	21,91198471	14,77160308
2F4.12	ARNG2	T3	Heterozygote	(-G), position 14	WT	Heterozygote		(-TCCTCGGGTG), position7	(-G), position 14	Homozygote		WT	WT	WT		-/1,-/-,3/3	25,86443975	14,57771521
2F4.13	ARNG2	T3	Heterozygote	WT	WT	WT		(-TCCTCGGGTG), position7	WT	Heterozygote		(-G), position 14	WT	Heterozygote		1/1,-/2,-/3	20,66636978	11,47321936
2F4.14	ARNG2	T3	Heterozygote	(-G), position 14	WT	Heterozygote		(-TCCTCGGGTG), position7	(-TCCTCGGGTG), position7	Homozygote		WT	WT	WT		-/1,-/-,3/3	27,71374584	16,08196101
2F4.15	ARNG2	T3	Heterozygote	WT	WT	WT		(-TCCTCGGGTG), position7	WT	Heterozygote		(-G), position 14	WT	Heterozygote		1/1,-/2,-/3	20,08690309	11,70193224
2F4.16	ARNG2	T3	Homozygote	WT	WT	WT		(-TCCTCGGGTG), position7	WT	Heterozygote		(-TG), position 15	(-TG), position 15	Homozygote		1/1,-/2,-/-	23,54652933	15,1057756
2F4.17	ARNG2	T3	Homozygote	(-G), position 14	WT	Heterozygote		(-TCCTCGGGTG), position7	WT	Heterozygote		(-G), position 14	WT	Heterozygote		-/1,-/2,-/3	22,7717816	13,54144907
2F4.18	ARNG2	T3	Homozygote	(-G), position 14	WT	Heterozygote		(-TCCTCGGGTG), position7	(-TCCTCGGGTG), position7	Homozygote		(-G), position 14	WT	Heterozygote		-/1,-/-,-/3	43,80650689	29,92858727
2F4.19	ARNG2	T3	Homozygote	WT	WT	WT		(-TCCTCGGGTG), position7	(-G), position 14	Homozygote		(-G), position 14	WT	Heterozygote		1/1,-/-,-/3	25,92364865	13,11997041
2F4.20	ARNG2	T3	Heterozygote	WT	WT	WT		(-TCCTCGGGTG), position7	WT	Heterozygote		(-TG), position 15	(-TG), position 15	Homozygote		1/1,-/2,-/-	24,08996348	13,30411858
2F4.21	ARNG2	T3	Heterozygote	(-G), position 14	(-G), position 14	Homozygote		(-TCCTCGGGTG), position7	(-TCCTCGGGTG), position7	Homozygote		WT	WT	WT		-/-,-/-,3/3	34,90473713	24,03277779
2F4.22	ARNG2	T3	Homozygote	(-G), position 14	WT	Heterozygote		(-TCCTCGGGTG), position7	WT	Heterozygote		(-G), position 14	(-G), position 14	Homozygote		-/1,-/2,-/-	33,34035101	20,00273075
2F4.23	ARNG2	T3	Heterozygote	(-G), position 14	(-G), position 14	Homozygote		(-G), position 14	(-G), position 14	Homozygote		WT	WT	WT		-/-,-/-,3/3	35,9445815	20,40726575
2F4.24	ARNG2	T3	Heterozygote	WT	WT	WT		(-TCCTCGGGTG), position7	(-TCCTCGGGTG), position7	Homozygote		(-G), position 14	(-TG), position 15	Homozygote		1/1,-/-,-/-	40,62882134	17,95412435
2F4.25	ARNG2	T3	Heterozygote	WT	WT	WT		(-TCCTCGGGTG), position7	(-TCCTCGGGTG), position7	Homozygote		(-G), position 14	WT	Heterozygote		1/1,-/-,-/3	28,4763654	13,21234132
2F4.27	ARNG2	T3	Homozygote	(-G), position 14	WT	Heterozygote		(-TCCTCGGGTG), position7	WT	Heterozygote		(-G), position 14	WT	Heterozygote		-/1,-/2,-/3	27,35321311	15,3533615
2F4.28	ARNG2	T3	Heterozygote	(-G), position 14	(-G), position 14	Homozygote		WT	WT	WT		(-G), position 14	WT	Heterozygote		-/-,2/2,-/3	20,38441168	14,93579676
2F4.29	ARNG2	T3	Homozygote	(-G), position 14	(-G), position 14	Homozygote		(-G), position 14	(-G), position 14	Homozygote		(-G), position 14	WT	Heterozygote		-/-,-/-,-/3	52,91731329	33,6873005
2F4.30	ARNG2	T3	Heterozygote	(-G), position 14	WT	Heterozygote		(-TCCTCGGGTG), position7	WT	Heterozygote		(-TG), position 15	(-TG), position 15	Homozygote		-/1,-/2,-/-	52,693978	19,48449904
