# Supplementary material for: Selective gene dosage by CRISPR‐Cas9 genome editing in hexaploid Camelina sativa
Source: Plant Biotechnol J. 2017 Apr 1;15(6):729–39. doi: 10.1111/pbi.12671 (PMC5425392; doi:10.1111/pbi.12671)
Supplement: Supplementary file 3 — Supplementary Caption [file PBI-15-729-s003.docx]

**Supplementary information**

**Figure S1.** Structure of Camelina and Arabidopsis *FAD2* genes.

(A) Arabidopsis *FAD2* coding sequence and protein translation. Highlighted in purple, ER localization signal; blue, metal ion binding putative catalytic site and grey, putative transmembrane domains. The two sgRNA1 and sgRNA2 sequence are indicated respectively in green and blue with PAM site underlined.

(B) Camelina *CsFAD2* coding sequences and protein translation surrounding the sgRNA target sites.

(C) Sequences of the sgRNA cassettes. AttB sites used for cloning of the fragment in pDONR207 are indicated in blue, the camelina U3 and U6 promoter are in green and orange respectively, the a and g nucleotides, important for optimal transcription (Cong et al., 2013; Shan et al., 2013) are in yellow, the 20bp targets are in red and the tracrRNA is in black.

For figures B and C, the sequences in green and blue show respectively sgRNA1 and sgRNA2 with the PAM site underlined and in red are indicated Camelina v. Celine polymorphisms compared to sequence database.

**Figure S2.** Camelina *CsFAD2* coding sequences and the different primers used in the study. The two sgRNA1 and sgRNA2 sequence are indicated respectively in green and blue and the sequence polymorphisms between the three *CsFAD2* genes are indicated in red.

**Figure S3.** Combinatorial *fad2* alleles associated with C18 content in T2 and T3 *CsFAD2* CRISPR lines.

(A) Distribution of *fad2* allele combinations at the *CsFAD2-1, CsFAD2-2* and *CsFAD2-3* loci in individual T2 leaves (bottom) and the resulting effect on the relative content of C18:0, C18:1, C18:2 and C18:3 of T3 seed progeny. Wild-type Camelina (v. Celine) are boxed in black and the T2 lines used to generate the T3 plants for further analysis are boxed in red.

(B) Distribution of *fad2* allele combinations at the *CsFAD2-1, CsFAD2-2* and *CsFAD2-3* loci in individual T3 leaves (bottom) and the resulting effect on the relative content of C18:0, C18:1, C18:2 and C18:3 of T3 leaves. Wild-type Camelina (v. Celine) are boxed in black.

(C) Distribution of *fad2* allele combinations at the *CsFAD2-1, CsFAD2-2* and *CsFAD2-3* loci in individual T3 leaves (bottom) and the resulting effect on the relative content of C18:0, C18:1, C18:2 and C18:3 of T4 seed progeny. Wild-type Camelina (v. Celine) are boxed in black.

Mutations (deletion/insertion) leading to sequence frameshift are indicated in red while those only associated with deletion or insertion without frameshift are indicated in purple.

**Figure S4.** OAI for the different allelic combinations at the three *CsFAD2* loci. Uppercase, WT allele. Blue lowercase, mutated allele. The number of lines used for each genetic combinations are indicated (n).

**Figure S5.** Expression levels of *CsFAD2* genes. Data were extracted from Camelina eFP browser (http://bar.utoronto.ca/efp_Camelina/cgi-bin/efpWeb.cgi). The data were collected from the published transcriptome (Kagale et al., 2016). The database was searched with the following accessions *Csa19g016350, Csa01g013220* and *Csa15g016000* for respectively *CsFAD2A, CsFAD2B* and *CsFAD2C*.

**Figure S6.** Fatty acid content of oil and cake fractions of selected *CsFAD2* CRISPR lines analyzed in Fig.2A and C. (A) Total fatty acid content of *CsFAD2* CRISPR lines. (B-C) Fatty acid profile of mechanically extracted oil (B) and the resulting cake (C).

**Figure S7.** Camelina U3 and U6 promoters used in this study. Sequences were identified by Basic Local Alignment Search Tool (http:// www.phytozome.net/physcomitrella_er.php) using the Arabidopsis U6-26 and U3B snRNA sequences (X52528 and X52629 respectively) as queries.

**Table S1.** Primer sequences used for amplification and sequencing. In red are indicated the nucleotide exchanges (new nucleotide: in red and nucleotide replaced : red in bracket) in order to generated a SNP. In bold black are indicated the SNP specific to the *CsFAD2* copies.

**Table S2.** Summary of the different genotypes of *CsFAD2* CRISPR T2 and T3 lines. DsRED indicates the fluorescence status of the seeds (negative, no fluorescence detected in seeds; Homozygous means 100% of the seeds are fluorescents; Heterozygous, segregating fluorescence). The sequence for both allele of each homeologous gene is indicated (WT, wild type sequence; +, indicates insertion and position compared to PAM site; - indicates deletion and position compared to PAM site) as well as the resulting genotype. The global genotype of the plant is indicated as follow : 1, 2 and 3 are wild type allele of respectively *CsFAD2-1, CsFAD2-2, CsFAD2-3* and – indicates a mutation in the corresponding allele. OAI index for T3 and T4 seeds as well as T3 leaves are shown. The symbol / indicates that data is not available.
